# Supplementary material for: Anti-gene oligonucleotides targeting Friedreich’s ataxia expanded GAA⋅TTC repeats increase Frataxin expression
Source: Mol Ther Nucleic Acids. 2025 Apr 17;36(2):102541. doi: 10.1016/j.omtn.2025.102541 (PMC12143624; doi:10.1016/j.omtn.2025.102541)
Supplement: Document S1. Figures S1–S7 and Table S1 [file mmc1.pdf]

OMTN, Volume *36*

Supplemental information

Anti-gene oligonucleotides targeting Friedreich's ataxia expanded GAA·TTC repeats increase Frataxin expression

Negin Mozafari, Salomé Milagres, Tea Umek, Cristina S.J. Rocha, Claudia M. Vargiu, Fiona Freyberger, Osama Saher, Marek Napierala, Jill S. Napierala, Pontus Blomberg, Per T. Jørgensen, Tanel Punga, C. I. Edvard Smith, Jesper Wengel, and Rula Zain

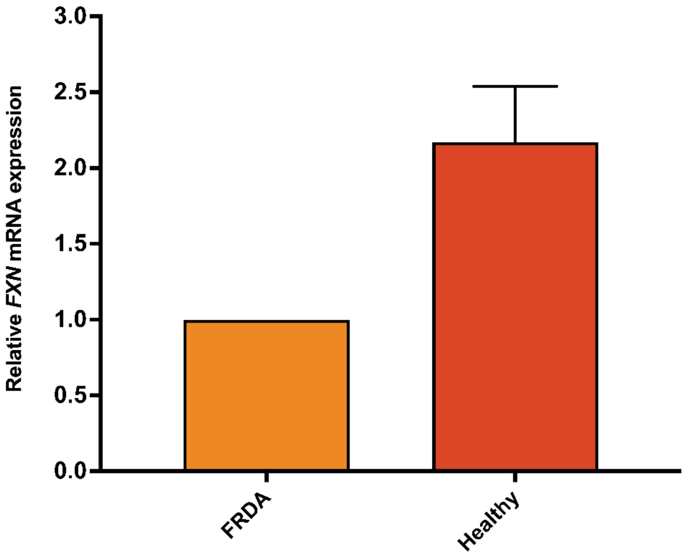

Figure S1. *FXN* mRNA levels of GM03816 (FRDA) and GM08402 (healthy) fibroblasts were assessed by RT-qPCR. The values were normalized to *HPRT1* levels as reference gene and the level of *FXN* mRNA in FRDA cells was set to one. Results are presented as Mean ±SD (n=3). Difference between means (healthy-FRDA) ± SD =1.171 ± 0.2117.

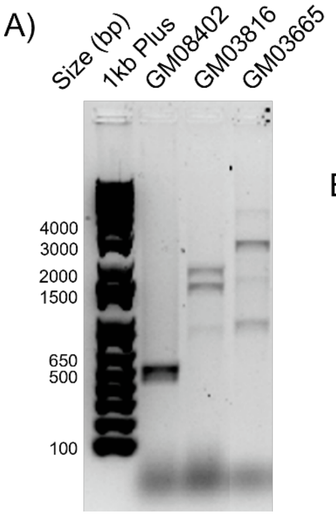

B)

Cell line	Repeats	Expected bp	Expected bp + flanking region
**GM08402**	Healthy	-	“580-580”
**GM03816**	330-380	990-1140	1570 - 1720
**GM03665**	780-1410	2340-4230	2920-4900

Figure S2. *FXN* gene GAA•TTC repeat length in primary fibroblasts used in this study. Genomic DNA was extracted from GM08402, GM03816 and GM03665 patient derived fibroblasts and the GAA•TTC repeats at the *FXN* locus were amplified by PCR. **A)** Agarose gel analysis of GAA•TTC repeat size in GM08402 (healthy) and GM03816 and GM03665 (FRDA) fibroblasts. **B)** GAA•TTC repeat length and expected size after PCR amplification.

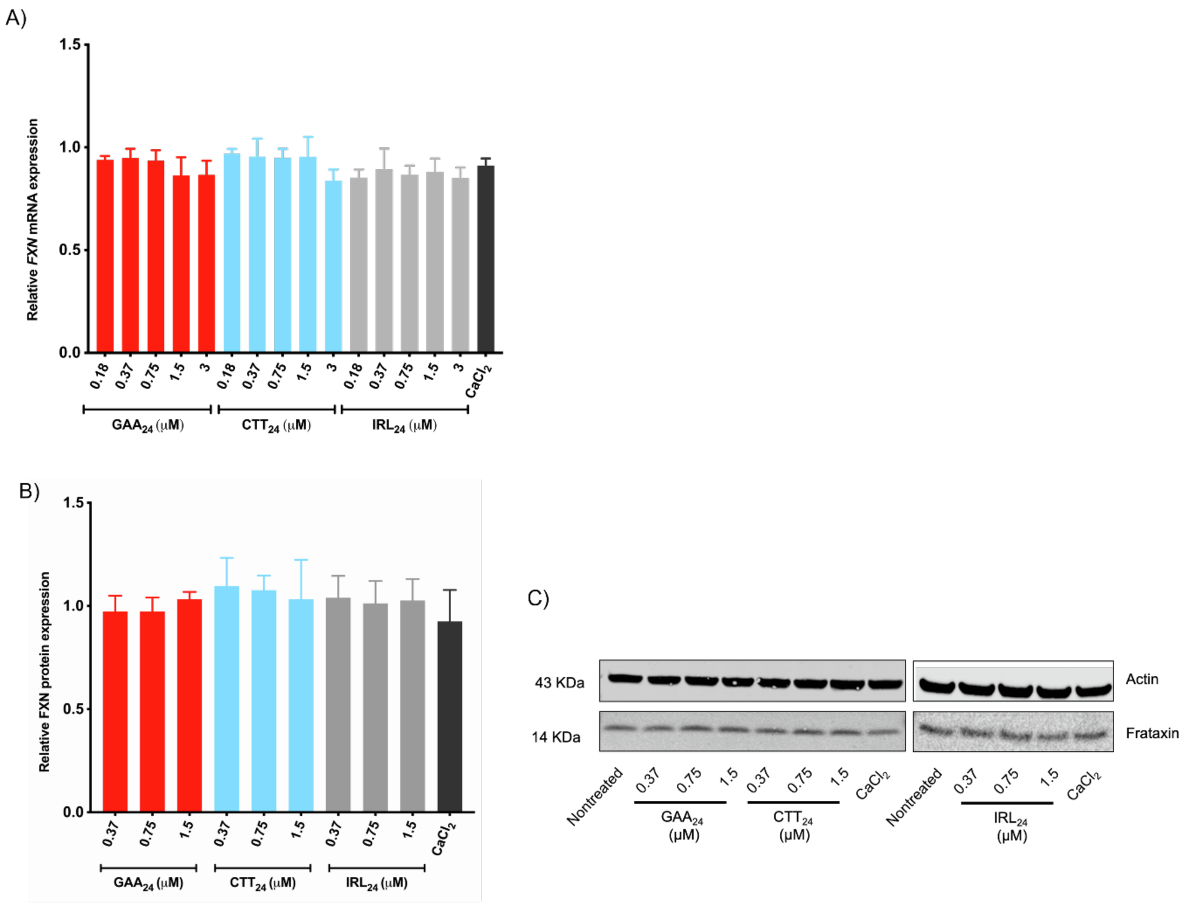

Figure S3. A-GOs do not affect neither FXN mRNA nor protein expression in healthy fibroblasts. FXN mRNA and protein expression of 6718 healthy fibroblasts after gymnotic delivery of ONs in medium supplemented with 9 mM $CaCl_2$. The cells were harvested 4 days post-treatment. *FXN* mRNA levels were determined using RT-qPCR and *FXN* levels were normalized to *HPRT1* as a reference gene. FXN protein levels were determined using western blot and FXN was normalized to Actin levels as a reference gene. For both mRNA and protein expression, relative FXN expression is shown after normalizing to the control NT cells. Results are presented as Mean ±SD, n≥3. Statistics were performed with one-way ANOVA Multiple Comparison, (Šidák), towards control ONs. (* = $P < 0.05$, ** = $P < 0.01$, *** = $P < 0.001$).

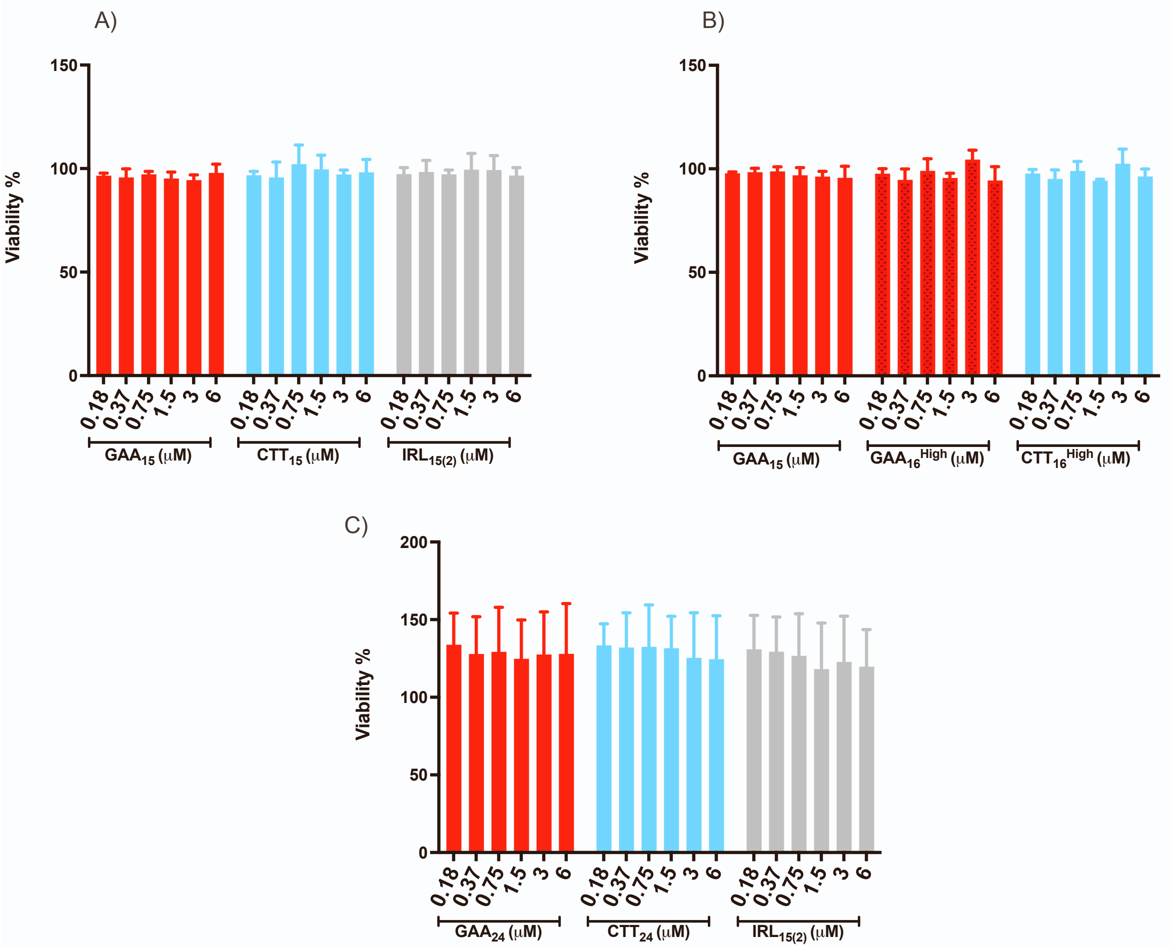

Figure S4. No significant cytotoxicity was detected after treatment of FRDA derived patient's cells with selected ONs. Viability percentage of GM03816 cells after gymnotic delivery of ONs in medium supplemented with 9 mM $CaCl_2$ throughout 48 hours treatment. The relative values were obtained by normalization of ONs treated cells versus cells in the presence of 9 mM $CaCl_2$. Results are presented as Mean ±SD, A and B (n=3) and C (n=2). Statistics were performed with two-way ANOVA Multiple Comparison (Turkey). The mean of each condition was compared to the mean of every other condition. (* = $P < 0.05$, ** = $P < 0.01$, *** = $P < 0.001$).

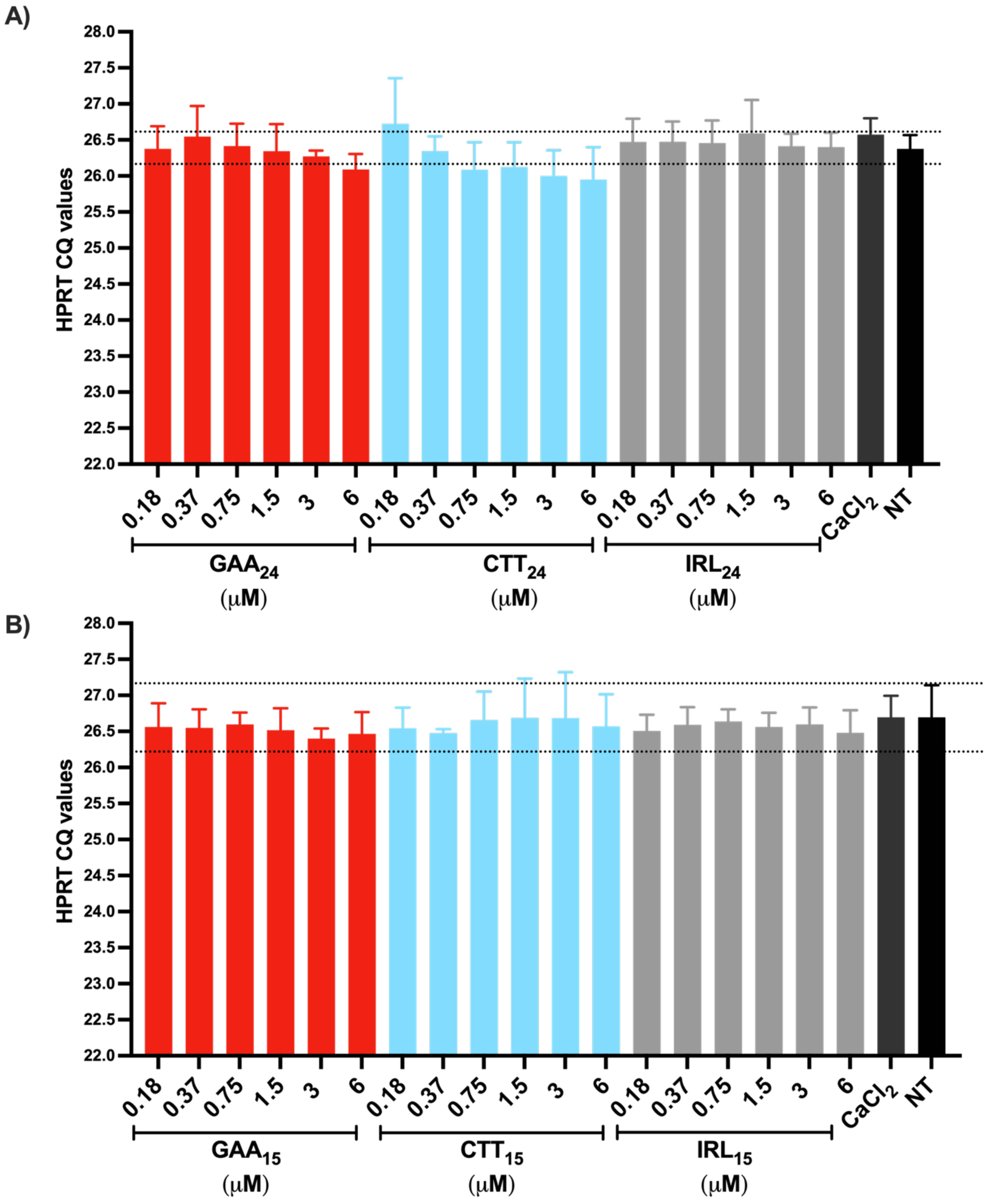

Figure S5. No significant differences in *HPRT1* C_q values were detected after treatment of FRDA derived patient's cells with selected ONs. GM03816 fibroblasts were treated with **A)** 15-mers and **B)** 24-mers GAA, CTT and IRL ONs at different doses ranging from 0.18 - 6 µM. Treated and NT cells were harvested 4 days after transfection, and *HPRT1* C_q values were assessed by RT-qPCR. Results are presented as Mean ±SD (n≥3). Statistics were performed with Kruskal-Wallis test, towards NT cells.

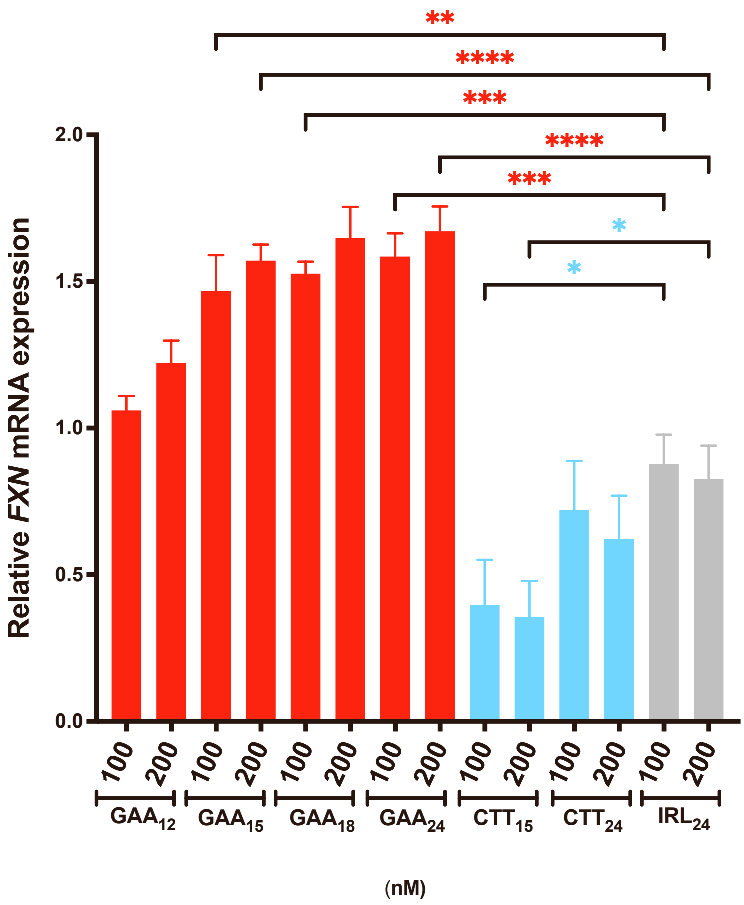

Figure S6. GAA A-GOs upregulate *FXN* mRNA expression in a FRDA cell model with a higher number of repeats. GM03665 fibroblasts were treated with different lengths of GAA, CTT and IRL ONs at 100 and 200 nM. Treated and NT cells were harvested 4 days after transfection, and *FXN* mRNA levels were assessed by RT-qPCR. The values were normalized to *HPRT1* levels as reference gene and were compared to NT cells. Results are presented as Mean with SD ($n \geq 3$). Statistics were performed with one-way ANOVA Multiple Comparison, (Šídák), towards corresponding IRL concentration. (* = $P < 0.05$, ** = $P < 0.01$, *** = $P < 0.001$, **** = $P < 0.0001$;).

A)

Gene	Symbol	Repeats (location; number)	Binding of GAA_{24}
Adenosine Kinase	ADK	GAA:TTC (intron 6; 13)	DNA template strand
Ribosomal protein S6 kinase A5	RPS6KA5	AAG:CTT (3'UTR; 8)	DNA template strand
Lysine acetyltransferase 6B	KAT6B	GAA:TTC (exon 16; 8)	DNA template strand
PR/SET domain 10	PRDM10	CTT:AAG (3'UTR; 10)	mRNA
Ras And Rab Interactor 2	RIN2	CTT:AAG (intron 2; 22 & 14)	pre-mRNA

B)

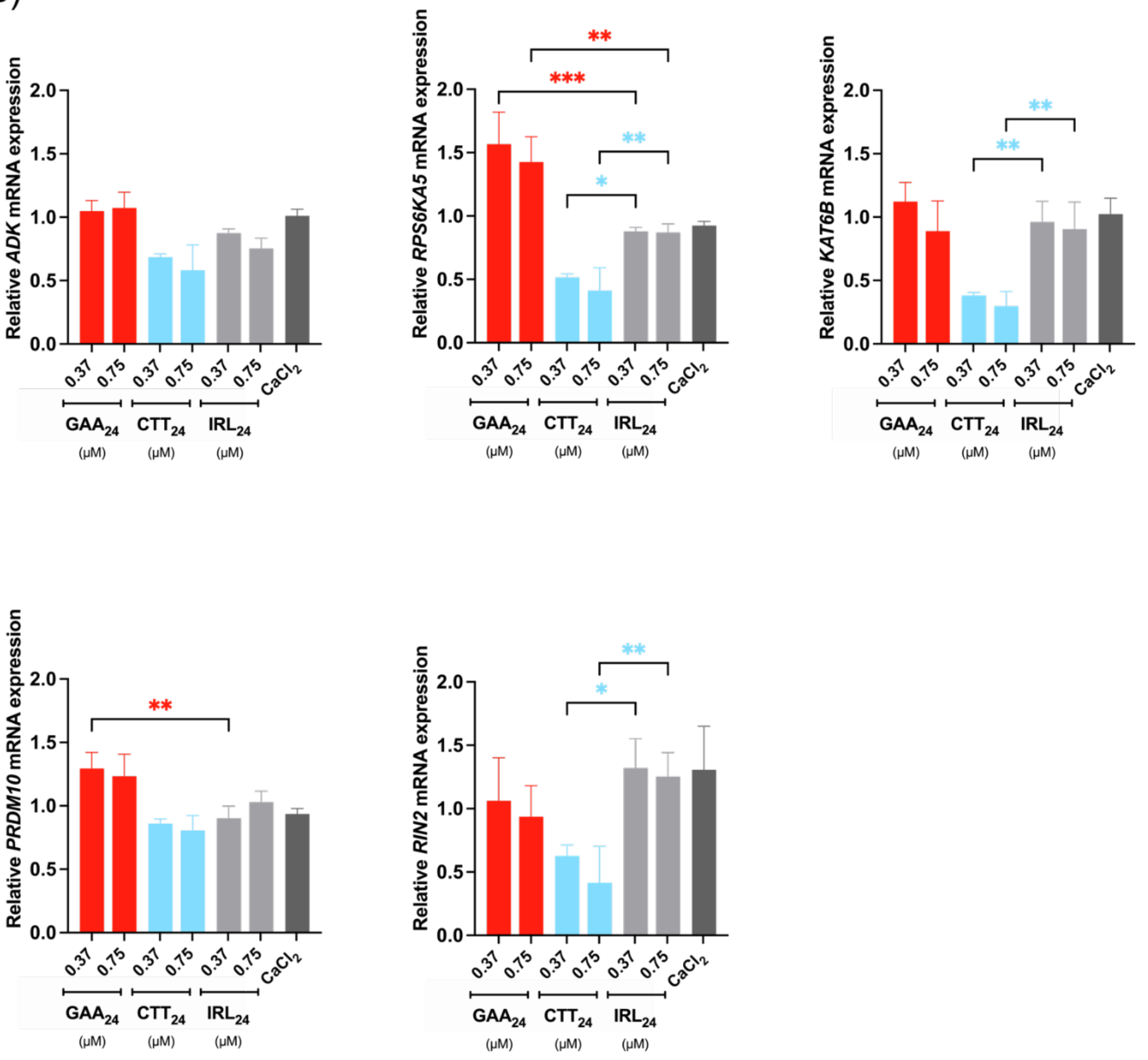

Figure S7. GAA_{24} and CTT_{24} affect the mRNA levels of other repeat-containing genes. A) Information on the selected genes for off-target analysis. **B)** 4869 fibroblasts were treated with concentrations of 0.37 and 0.75 µM, in medium supplemented with 9 mM $CaCl_2$. Four days post-treatment, the cells were harvested, and mRNA levels were analysed. The values were normalized to *HPRT* as a reference gene. The expression of NT cells without the presence of $CaCl_2$ was set to 1. Results are presented as Mean ± SD, (n = 3). Statistics were performed with one-way ANOVA Multiple Comparison (Šidák) towards the same IRL_{24} concentration (* = $P < 0.05$, ** = $P < 0.01$, *** = $P < 0.001$).

Table S1. List of all the primers and probe sets used in this study. *FXN* was multiplexed with *HPRT1*_v1, *RPS6KA5* to *KAT6B* were multiplexed with *HPRT1*_v2 and *RIN2* was multiplexed with *HPRT1*_v3. *HPRT1*_v1, *FXN* and *HPRT1*_v3 sets were purchased as DNA oligos and dual-labelled probes from Merck. *HPRT1*_v2, *RPS6KA5*, *PRDM10*, *ADK*, *KAT6B* and *RIN2* were purchased as PrimeTime™ qPCR Probe Assays from IDT. 6FAM, 56FAM, 5HEX and HEX are fluorophores. BHQ1, 3IABkFQ and ZEN are quenchers.

Gene	Sequence (5'-3')	
***HPRT1*_v1**	Fw primer	AGGGATTTGAATCATGTTTG
	Rv primer	CGATGTCAATAGGACTCC
	Probe	/6FAM/ACTCAACTTGAACTCTCATCTTAGGCT/BHQ1/
FXN	Fw primer	GTGGAGATCTAGGAACCTATG
	Rv primer	TTAAGGCTTTAGTGAGCTCTG
	Probe	/HEX/TCCAGTCATAACGCTTAGGTCCAC/BHQ1/
***HPRT1*_v2**	Fw primer	AGGATTTGGAAAGGGTGTTTATTC
	Rv primer	CCCATCTCCTTCATCACATCTC
	Probe	/56-FAM/ATGGACAGG/ZEN/ACTGAACGTCTTGCT/3IABkFQ/
RPS6KA5	Fw primer	CATGCTGAGAAGGTGGGAATAG
	Rv primer	GGCATACAGCTTTCCAGTATCA
	Probe	/5HEX/TTGAGCTCC/ZEN/TGAAGGTCCTAGGAACT/3IABkFQ/
PRDM10	Fw primer	GGACACAGGACTTGCTACATT
	Rv primer	GGATTGGGATAGTGGTCTGTTC
	Probe	/5HEX/ACTCTGAAT/ZEN/GGGCTGGATCAACCA/3IABkFQ/
ADK	Fw primer	GCTGAAGACAAACACAAGGAAC
	Rv primer	TCATCCACTGAGCCACTTTAAT
	Probe	/5HEX/ATGCTGGTG/ZEN/GCTCTACCCAGAATT/3IABkFQ/
KAT6B	Fw primer	GACAAACAGAGGAAGAGGAAGG
	Rv primer	CACTTTAGAGGTTCGGGATTGT
	Probe	/5HEX/TGCTTCAAG/ZEN/AATGCTGACCCTTGTAGA/3IABkFQ/
***HPRT1*_v3**	Fw primer	GAGCTATTGTAATGACCAGTC
	Rv primer	TGACCAAGGAAAGCAAAG
	Probe	/6FAM/TGCCAGTGTCAATTATATCTTCCACAA/BHQ1/
RIN2	Fw primer	CCACTCAAGGAATTTGCCATAAA
	Rv primer	GAAAGCAATGAGCCGGAATAAA
	Probe	/5HEX/CCCTGGAAG/ZEN/GCTCAGGAATCAGTTT/3IABkFQ/
