# Supplementary material for: Anti-gene oligonucleotides targeting Friedreich’s ataxia expanded GAA⋅TTC repeats increase Frataxin expression
Source: Mol Ther Nucleic Acids. 2025 Apr 17;36(2):102541. doi: 10.1016/j.omtn.2025.102541 (PMC12143624; doi:10.1016/j.omtn.2025.102541)
Supplement: Document S2. Article plus supplemental information [file mmc2.pdf]

Molecular Therapy
Nucleic Acids
Original Article

Anti-gene oligonucleotides targeting Friedreich's ataxia expanded GAA•TTC repeats increase Frataxin expression

Negin Mozafari,[1,2,10] Salomé Milagres,[1,2,10] Tea Umek,[1,2,3] Cristina S.J. Rocha,[1,9] Claudia M. Vargiu,[1] Fiona Freyberger,[1] Osama Saher,[1,4] Marek Napierala,[5] Jill S. Napierala,[5] Pontus Blomberg,[1,6] Per T. Jørgensen,[7] Tanel Punga,[3] C. I. Edvard Smith,[1,2] Jesper Wengel,[7] and Rula Zain[1,2,8]

[1]Department of Laboratory Medicine, Karolinska Institutet, ANA Futura, Alfred Nobels Allé 8, SE-141 52 Huddinge, Stockholm, Sweden; [2]Karolinska ATMP Center, Karolinska Institutet, Karolinska University Hospital, SE-171 76 Stockholm, Sweden; [3]Department of Medical Biochemistry and Microbiology (IMBIM), Uppsala University, 75123 Uppsala, Sweden; [4]Department of Pharmaceutics and Industrial Pharmacy, Faculty of Pharmacy, Cairo University, Cairo, Egypt; [5]Department of Neurology, O'Donnell Brain Institute, University of Texas Southwestern Medical Center, Dallas, TX, USA; [6]Karolinska Cell Therapy Center, Karolinska University Hospital, Stockholm, Sweden; [7]Department of Physics, Chemistry and Pharmacy, Biomolecular Nanoscale Engineering Center, University of Southern Denmark, Odense, Denmark; [8]Center for Rare Diseases, Clinical Genetics and Genomics, Karolinska University Hospital, SE-17176 Stockholm, Sweden

Friedreich's ataxia is a progressive, autosomal recessive ataxia caused, in most cases, by homozygous expansion of GAA•TTC triplet-repeats in the first intron of the *Frataxin* gene. GAA•TTC repeat expansion results in the formation of a non-B-DNA intramolecular triplex as well as changes in the epigenetic landscape at the *Frataxin* locus. Expansion of intronic GAA•TTC repeats is associated with reduced levels of *Frataxin* mRNA and protein, resulting in disease development. In our previous study, we demonstrated that DNA-binding anti-gene oligonucleotides specifically targeting the GAA•TTC repeat expansion effectively disrupted the formation of intramolecular triplex structures. In this study, we extend these findings by showing that targeting repeat-expanded chromosomal DNA with anti-gene oligonucleotides leads to an increase in *Frataxin* mRNA and protein levels in cells derived from Friedreich's ataxia patients. We examined numerous anti-gene oligonucleotides and found that the design, length, and their locked nucleic acid composition have a high impact on the effectiveness of the treatment. Collectively, our results demonstrate the unique capability of specifically designed oligonucleotides targeting the GAA•TTC DNA repeats to upregulate *Frataxin* gene expression.

INTRODUCTION

Friedreich's ataxia (FRDA) is a rare, inherited disorder that causes progressive damage to the nervous system. Additionally, the peripheral nervous system, heart muscle, skeleton, and pancreas are also affected.[1] The patients normally begin to show symptoms during childhood or the first years of adolescence and lose their walking ability on average 10 to 15 years after disease onset.[2,3] FRDA is the most common inherited ataxia, with a prevalence of about 1 in 50,000 individuals. It is primarily caused by homozygous GAA•TTC triplet-repeat expansion within the first intron 1 of the *Frataxin* (*FXN*) gene. Importantly, homozygous expansion of the GAA•TTC triplet-repeats in the *FXN* gene is associated with FRDA in 98% of affected individuals.[4–6]

The *FXN* gene encodes the FXN protein, primarily localized in mitochondria, where it plays a key role in iron homeostasis and mitochondrial function. Post-maturation, FXN can also be found in other cellular locations such as the nucleus, endoplasmic reticulum, and microsomes, indicating a broader range of cellular roles beyond the mitochondria.[7,8] Notably, the FXN protein is responsible for the iron-sulfur (Fe-S) cluster biosynthesis, acting as an allosteric activator.[9] The role of Fe-S clusters in cells is diverse, from electron transfer and Fe ion regulation to DNA repair. Malfunctioning of these processes causes Fe-S cluster deficiency and accumulation of toxic iron in mitochondria.[10] The expansion of GAA•TTC repeats in the *FXN* gene results in reduced transcription and subsequently lowered levels of FXN protein, leading to increased mitochondrial oxidative stress and cellular damage.[10–12]

In FRDA, the length of the GAA•TTC repeats is associated with the age of disease onset and severity of disease. Pathogenic expanded alleles carry 66–1,700 GAA•TTC repeats, whereas an unaffected allele contains 7 to 22.[13,14] Haploinsufficiency in individuals with an expanded GAA•TTC repeat on one allele, while the other allele has an unaffected repeat number, results in approximately 50% reduction of FXN levels. However, these individuals typically remain

Received 25 September 2024; accepted 14 April 2025;
https://doi.org/10.1016/j.omtn.2025.102541.

[9]Present address: ESS, Polytechnic of Porto, Rua Dr. António Bernardino de Almeida, 4200-072 Porto, Portugal
[10]These authors contributed equally
Correspondence: Rula Zain, LabMed, Karolinska Institutet, SE-141 52 Stockholm, Sweden.
E-mail: rula.zain@ki.se

© 2025 The Authors.
Published by Elsevier Inc. on behalf of The American Society of Gene and Cell Therapy.
This is an open access article under the CC BY license (http://creativecommons.org/licenses/by/4.0/).

asymptomatic, indicating that the reduced FXN levels are still sufficient for maintaining cellular function.[6,15,16]

Germline instability of expanded GAA•TTC repeats occurs during both paternal and maternal transmission. Apart from the intergenerational unstable transmission, the expansion of GAA•TTC repeats varies extensively within the individual's tissues.[17,18] Somatic instability of pathogenic expanded alleles is progressive throughout lifetime,[19] affecting tissues like the heart, dorsal root ganglia, cerebellum, pancreas, and spinal cord.[17,18]

Several factors are reported to cause disturbed transcription initiation or elongation, thereby contributing to *FXN* gene silencing in FRDA.[14,20–24] It has been reported that expanded GAA•TTC repeats form non-B-DNA structures like intramolecular triplex conformations (H-DNA) or DNA-RNA structures (R-loop), reducing *FXN* mRNA and hence also protein levels.[14,20,21] Furthermore, epigenetic changes and heterochromatin formation are linked to *FXN* gene silencing at the expanded locus.[22] Two isoforms of H-DNA have been proposed to form at GAA•TTC repeats by chemical probing *in vitro*; the purine (YR•R) and the pyrimidine (YR•Y) motif triplex.[25] However, there is conflicting *in vitro* evidence regarding the predominant H-DNA isoform.[26,27] Recently, an S1-END-seq assay was used to examine triplex formation at the *FXN* locus *in vivo* and identified two H-DNA isoforms at homopurine/homopyrimidine-rich repeats across the genome.[28]

Currently, there is no cure available for FRDA, and the existing therapies only treat symptoms.[2,29] In February 2023, the Food and Drug Administration approved omaveloxolone (Skyclarys), making it the first and only approved drug for FRDA. Omaveloxolone functions by activating nuclear factor erythroid 2-related factor 2 (Nrf2), a transcription factor that is suppressed in FRDA. Nrf2 plays a crucial role in maintaining redox homeostasis and mitigating the production of reactive oxygen species.[30] Additional trials are ongoing and comprise a variety of disease-modifying substances targeting pathways underlying FRDA pathogenesis.[7] Thus, derivatives of 2-aminobenzamide and nicotinamide upregulate FXN expression in both patient-derived cells and patients.[31–33] Other strategies include histone deacetylase inhibitors (HDACi), CRISPR-Cas9 technology, and AAV-based gene delivery.[33,34]

Most recently, antisense oligonucleotides (ASOs) directed against the 5′ and 3′ untranslated region (UTR) of *FXN* mRNA were shown to increase FXN mRNA and protein expression in FRDA patient-derived cell lines.[35] Moreover, increased FXN mRNA and protein expression has been achieved in cell culture by targeting the proposed R-loop with ASOs and duplex RNAs.[36,37] Nevertheless, the effect of these gapmer and steric block ASOs and also single-strand small interfering RNA (ss-siRNA) in cell cultures was not translatable in a FRDA mouse model.[38]

Our approach is to address the root cause of the disease, which is the expanded repeats. To this end, we have previously designed repeat-targeting anti-gene oligonucleotides (A-GOs) and demonstrated that our A-GOs abolished H-DNA formation at GAA•TTC expanded repeats in plasmids in a sequence- and structure-specific manner.[39,40] More recently, we showed that our A-GO modality, targeting the H-DNA at the GAA•TTC expanded repeats, prevented repeat expansion in mammalian cells in a plasmid-based reporter system.[41] Here we studied A-GO-mediated activation of *FXN* expression in FRDA patient-derived primary fibroblasts using locked nucleic acid/DNA (LNA/DNA) mixmers with a fully phosphorothioate (PS)-modified backbone targeting the H-DNA structure in the first intron of the *FXN* gene. We also evaluated the sequence design of LNA/DNA mixmers and the LNA content, and we observed that these parameters are key to achieving efficient *FXN* upregulation.

RESULTS

GAA A-GOs significantly enhance *FXN* mRNA expression

We have previously showed that modified GAA DNA-targeting A-GOs significantly prevented H-DNA triplex structure formation in plasmids containing pathogenic expanded $(GAA{\bullet}TTC)_{115}$ repeats while, conversely, CTT oligonucleotides (ONs) enhanced triplex formation.[39,40] We refer to GAA ONs as GAA A-GOs because they target *FXN* chromosomal DNA due to the absence of the complementary CTT sequence in pre-mRNA. In contrast, CTT ONs are referred to as ONs because they can act on both chromosomal DNA and pre-mRNA containing the GAA sequence. We hypothesized that GAA A-GOs could, by blocking the formation of H-DNA, facilitate *FXN* transcription and restore mRNA and protein levels (Figure 1A). To test this hypothesis, GAA A-GOs were designed as LNA/DNA mixmers with a fully PS-modified backbone (Figures 1B and 1C) and directed against the GAA•TTC repeats in FRDA patient-derived fibroblasts. First, we designed and synthesized various GAA A-GO mixmers of different lengths and LNA content (Table 1). Likewise, the corresponding CTT LNA/DNA mixmers were designed to be complementary to the released, single-strand chromosomal GAA region of the H-DNA (YR•Y) or to the pre-mRNA (Figures 1A–1C and Table 1).

To evaluate the activity of GAA A-GOs and CTT ONs on *FXN* expression, FRDA female patient fibroblasts, carrying approximately 330/380 GAA•TTC repeats (GM03816), were transfected with 200 nM of ONs. As shown in Figure 1D, only the GAA_{15} A-GO significantly upregulated *FXN* mRNA expression when compared with the control, $Scrambled_{19}^{High}$ ON. Two other 15-mer GAA A-GOs (GAA_{15}^{DNAEnd} and GAA_{15}^{LNAEnd}) were studied. They both contain DNA "adenosine" instead of LNA "adenosine" in the third position of the triplet and terminate with either a DNA or a LNA base. This comparison was performed to check whether these alterations impacted on the A-GO's efficiency, and they showed no significant effect on *FXN* mRNA expression (Figure 1D). Moreover, keeping the same design as GAA_{15}^{LNAEnd}, and increasing the GAA A-GO length from 15 to 16 (GAA_{16}^{Low}) or 19 (GAA_{19}^{Low}) did not improve A-GO activity (Figure 1D). Notably, the CTT ONs with the LNA positioning as the GAA A-GOs, significantly reduced *FXN* expression (Figure 1D). We hypothesize that the inclusion of additional

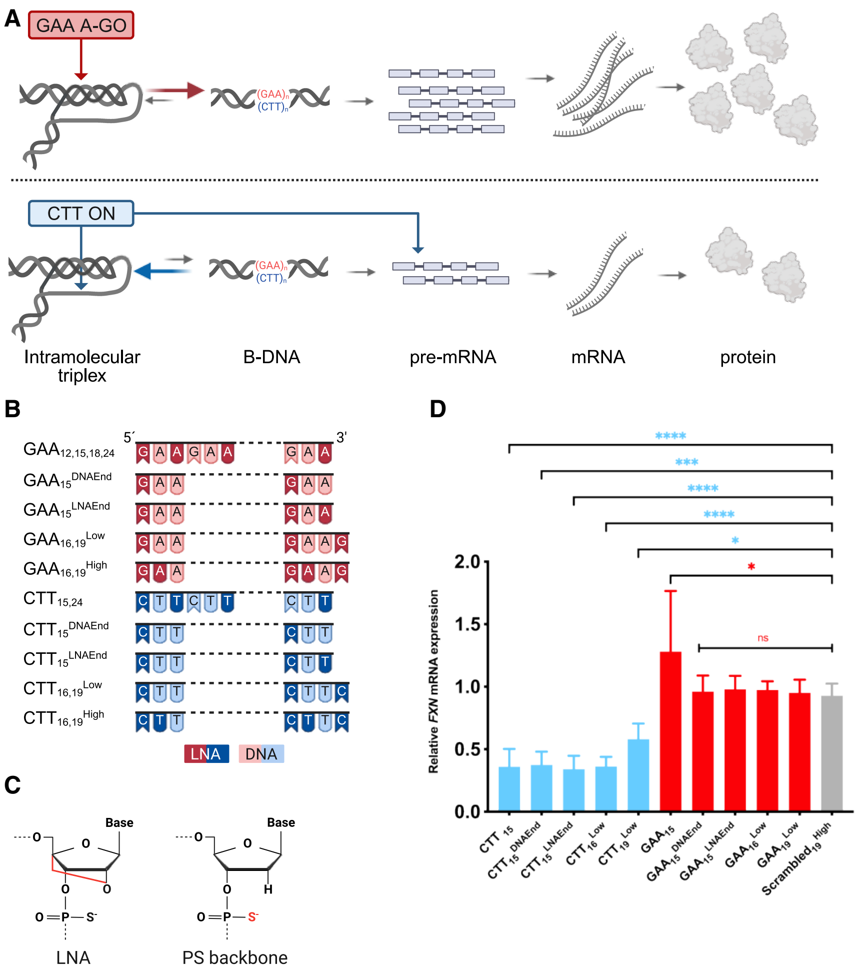

Figure 1. LNA composition influences the effect of GAA and CTT ONs on *FXN* mRNA expression

(A) Schematic representation of GAA and CTT ON interactions with the *FXN* gene and their proposed outcomes. (B) Illustrations of different GAA and CTT ONs used in this study. LNA bases are darker with white text, and DNA bases are in a lighter color with black text. (C) Chemical structures of the nucleic acid modifications used in this study. (D) Female FRDA patient-derived fibroblasts carrying 330/380 GAA•TTC repeats (GM03816) were treated with 200 nM GAA, CTT and IRL ONs, and *FXN* mRNA levels were analyzed with RT-qPCR 4 days after transfection. Relative *FXN* expression of each treatment was compared with nontreated (NT) cells and normalized to the ratio of the *FXN* gene to *HPRT1*. Results are presented as mean ± SD (n = 4). Statistical analysis was performed using one-way ANOVA, multiple comparisons (Dunnett) (*p < 0.05, ***p < 0.001, ****p < 0.0001; ns = nonsignificant). (A), (B), and (C) were created in BioRender by M.N. (2025), https://BioRender.com/e45v370.

LNA modifications at the 5′ end of GAA_{15} enhances its hybridization efficiency compared with other tested 15-mer GAA A-GOs. This increased hybridization efficiency likely contributes to the greater potency of GAA_{15} in upregulating *FXN* expression. Moreover, the increased hybridization efficiency of GAA_{15} is hypothesized to arise from the specific pattern of LNA modifications. In GAA_{15}, all LNA-modified nucleotides are adenosine, except for the first guanosine, which is also LNA-modified. This contrasts with other tested 15-mer GAA A-GOs, where all LNA-modified nucleotides are guanosine. Because of adenosine's smaller size and reduced steric hindrance compared with guanosine, a better alignment and pairing with the complementary strand should be facilitated.[42] It is important to note that the melting temperatures of these A-GOs are in similar ranges, indicating that the observed differences in hybridization efficiency are not due to variations in thermal stability but rather the intrinsic properties of the LNA-modified adenosine nucleotides. This enhanced hybridization efficiency likely contributes to the greater potency of GAA_{15} in upregulating *FXN* expression.

We compared FRDA and healthy individual fibroblast cell lines regarding *FXN* mRNA expression in relation to GAA•TTC repeat expansions. Our results showed that healthy fibroblasts (GM08402) relatively expressed 2.17 fold *FXN* compared with the FRDA fibroblasts (GM03816) (Figure S1). Additionally, we confirmed the presence of expanded GAA•TTC repeats in the FRDA fibroblasts, while the healthy fibroblasts GM04802 do not carry these expansions (Figure S2).

Length and LNA content determine the efficiency of GAA A-GOs in upregulating *FXN* transcription

Subsequently, we tested if increasing the ON LNA content and length would alter *FXN* expression by promoting their GAA hybridizing capacity.[43,44] Therefore, ONs were designed and synthesized to contain 67% to 69% LNA (Table 1). To study their activity, 200 nM of GAA_{16}^{High}, GAA_{19}^{High}, CTT_{16}^{High}, CTT_{19}^{High}, and the corresponding control sequence, $IRL_{24(2)}$, were transfected into GM03816 cells followed by *FXN* expression analysis 4 days post-transfection. Notably, the ONs with 67%–69% LNA content were toxic at 200 nM, a concentration at which ONs with lower LNA content were not (not shown). Thus, the toxicity of GAA A-GOs with high LNA content makes them unsuitable as therapeutic candidates in FRDA.

Table 1. Oligonucleotides used in this study

Name	Sequence (5′–3′)	LNA (%)	Calculated Tm (°C)
GAA_{12}	G*a*A*g*a*A*g*a*A*g*a*A	40	40
GAA_{15}	G*a*A*g*a*A*g*a*A*g*a*A*g*a*A	40	50
GAA_{15}^{DNAEnd}	G*a*a*G*a*a*G*a*a*G*a*a*G*a*a	33	45
GAA_{15}^{LNAEnd}	G*a*a*G*a*a*G*a*a*G*a*a*G*a*A	40	48
GAA_{16}^{Low}	G*a*a*G*a*a*G*a*a*G*a*a*G*a*a*G	38	51
GAA_{16}^{High}	G*A*a*G*A*a*G*A*a*G*A*a*G*A*a*G	69	66
GAA_{18}	G*a*A*g*a*A*g*a*A*g*a*A*g*a*A*g*a*A	39	58
GAA_{19}^{Low}	G*a*a*G*a*a*G*a*a*G*a*a*G*a*a*G*a*a*G	37	59
GAA_{19}^{High}	G*A*a*G*A*a*G*A*a*G*A*a*G*A*a*G*A*a*G	68	79
GAA_{24}	G*a*A*g*a*A*g*a*A*g*a*A*g*a*A*g*a*A*g*a*A*g*a*A	38	76
CTT_{15}	C*t*T*c*t*T*c*t*T*c*t*T*c*t*T	40	44
CTT_{15}^{DNAEnd}	C*t*t*C*t*t*C*t*t*C*t*t*C*t*t	33	41
CTT_{15}^{LNAEnd}	C*t*t*C*t*t*C*t*t*C*t*t*C*t*T	40	44
CTT_{16}^{Low}	C*t*t*C*t*t*C*t*t*C*t*t*C*t*t*C	38	47
CTT_{16}^{High}	C*T*t*C*T*t*C*T*t*C*T*t*C*T*t*C	69	62
CTT_{19}^{Low}	C*t*t*C*t*t*C*t*t*C*t*t*C*t*t*C*t*t*C	37	62
CTT_{19}^{High}	C*T*t*C*T*t*C*T*t*C*T*t*C*T*t*C*T*t*C	68	80
CTT_{24}	C*t*T*c*t*T*c*t*T*c*t*T*c*t*T*c*t*T*c*t*T*c*t*T	38	68
$IRL_{15(1)}$	G*a*G*t*g*G*a*t*A*t*a*G*a*g*G	40	48
$IRL_{15(2)}$	C*t*C*a*t*C*a*c*C*t*a*C*a*t*A	40	46
$Scrambled_{19}$	g*A*c*G*A*c*G*A*c*G*A*c*G*A*c*G*A*c*G	63	82
IRL_{24}	C*t*C*a*t*C*a*c*C*t*a*C*a*t*A*c*a*T*a*c*T*c*t*A	38	68
$IRL_{24(2)}$	C*T*c*A*T*c*A*C*t*T*T*c*T*T*a*T*T*t*A*C*c*C*t*C	67	91

Chemical modifications are marked as follows: Uppercase: LNA, lowercase: DNA, and *: phosphorothioate backbone.

Next, we assessed if the GAA A-GO effect on *FXN* expression (Figure 1) depends on its length. Variants of $GAA_{12,15,18,24}$ were synthesized, maintaining 38%–40% of the LNA content (Table 1). For comparison, two different lengths of CTT ONs (CTT_{15} and CTT_{24}) with the same design features were also tested (Table 1). FRDA fibroblasts GM03816 were transfected with 200 nM of GAA A-GOs, or CTT ONs or their corresponding non-targeting counterparts ($IRL_{15(1)}$ and $IRL_{15(2)}$; all with 38%–40% LNA content). The *FXN* mRNA expression was enhanced in a GAA A-GO length-dependent manner, with the GAA_{24} A-GO causing a 1.8-fold increase in *FXN* mRNA expression when compared with non-targeting controls ($IRL_{(15)1}$ or $IRL_{(15)2}$) (Figure 2A). In contrast, increasing the CTT ON length did not further decrease *FXN* mRNA expression (Figure 2A). These data confirm the effectiveness of GAA A-GOs containing 38%–40% LNA content in upregulating *FXN* expression.

Next, we evaluated the potency of GAA A-GOs and CTT ONs on *FXN* expression by gymnotic delivery, which has been found to yield a good correlation between *in vitro* and *in vivo* ON activities.[45] For this purpose, ONs in a final concentration of 3 μM were added to the cells in a medium supplemented with 9 mM $CaCl_2$ to facilitate gymnotic delivery. As a control, the cells were treated only with a medium supplemented with 9 mM $CaCl_2$. Cells were harvested 4 days post-treatment, and *FXN* mRNA levels were determined using RT-qPCR. Contrary to expectations, the shorter GAA_{15} was more potent and significantly upregulated *FXN* expression at a higher concentration (3 μM) compared with GAA_{24} (Figure 2B). Moreover, while both CTT_{15} and CTT_{24} downregulated *FXN* mRNA, we observed no statistically significant difference between them (Figure 2B).

Longer GAA A-GOs are more effective at lower doses

Since the longer GAA A-GO showed a milder effect on *FXN* expression after gymnotic delivery (Figure 2B) when compared with the GAA A-GO transfected cells (Figure 2A), we aimed to optimize the gymnosis conditions and evaluate the corresponding shorter GAA A-GOs. To determine the concentration of the A-GOs with the highest possible effect without triggering toxicity, we performed a dose-dependent ON response study with gymnotic delivery in FRDA (GM03816) cells (Figures 3A–3C). Although we intended to conduct EC50/IC50 concentration studies, it was not possible because the effect between the lowest and highest dose was not linear, particularly for GAA A-GOs, due to their specific

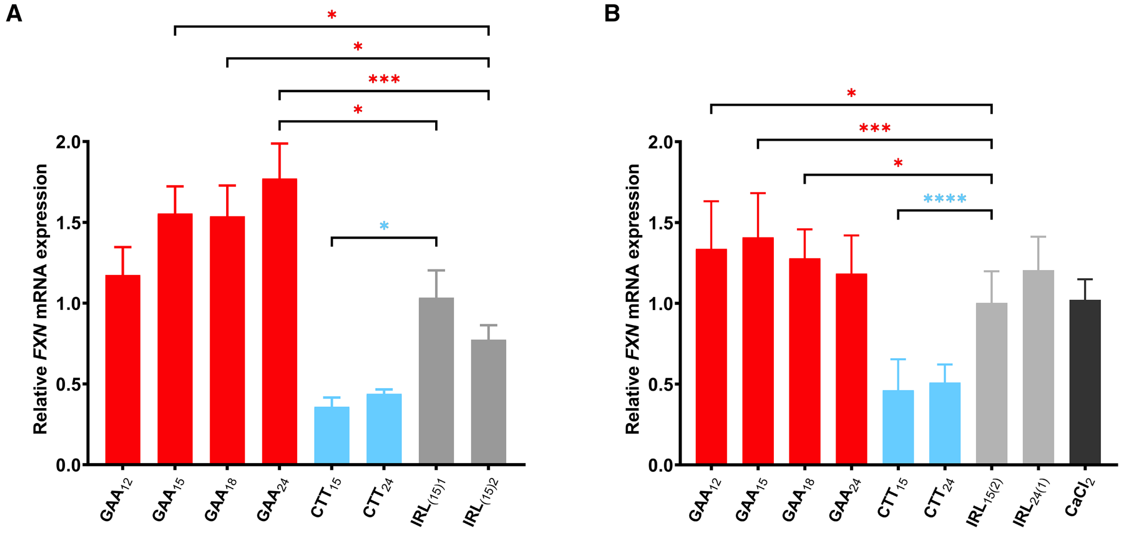

Figure 2. *FXN* upregulation improves with increasing GAA A-GO length at 200 nM transfection, but not at 3 μM gymnotic delivery

Female FRDA patient-derived fibroblasts carrying 330/380 GAA•TTC repeats (GM03816) were treated with (A) 200 nM GAA, CTT and IRL ONs, and *FXN* mRNA levels were analyzed with RT-qPCR 4 days after transfection. Relative *FXN* expression of each treatment was compared with NT cells and normalized to the ratio of the *FXN* gene to *HPRT1*. Results are presented as mean ± SD ($n = 3$). Statistics were performed with one-way ANOVA toward the corresponding random IRL ON consisting of the same LNA numbers and constitutions ($IRL_{15(1)}$). (B) 3 μM ONs in medium supplemented with 9 mM $CaCl_2$ were added to the cells the day after plating. After 4 days, total RNA was extracted, mRNA was quantified with RT-qPCR, and *FXN* levels were normalized to *HPRT1* and compared with NT cells. Results are presented as mean with SD ($n \geq 3$). Statistics were performed with one-way ANOVA multiple comparisons (Šidák) toward corresponding controls (*$p < 0.05$, **$p < 0.01$, ***$p < 0.001$).

mechanism of action. The highest and lowest ON concentrations varied 32-fold, ranging from 0.18 to 6 μM in medium supplemented with 9 mM $CaCl_2$.[45] For comparison, cells were treated with either non-targeting controls ($IRL_{15(2)}$ or IRL_{24}) or left untreated. Cells were harvested 4 days post-treatment, and *FXN* mRNA levels were determined using RT-qPCR. Interestingly, the GAA A-GOs of varying lengths showed different dose-response curves (Figure 3). For GAA_{15}, increasing the A-GO concentration to 6 μM translated to a more potent *FXN* mRNA upregulation (Figure 3A). For GAA_{18}, the effect was modest and seemed to plateau at concentrations above 0.75 μM (Figure 3B). In contrast, we observed that GAA_{24} was significantly more active at lower concentrations and displayed a maximum effect of 1.63-fold at 0.75 μM (Figure 3C). We then selected the optimal concentrations observed with GAA_{24} and tested them in a different FRDA cell model. While GM03816 fibroblasts originate from a female patient carrying approximately 330/380 GAA•TTC repeats, 4869 fibroblasts originate from a male patient carrying a similar number of repeats (approximately 294/405 GAA•TTC repeats). Similar to GM03816, 4869 fibroblasts were harvested 4 days after the treatment and *FXN* mRNA levels were determined using RT-qPCR. Both concentrations tested, 0.37 and 0.75 μM, significantly upregulated *FXN* expression up to 1.25-fold (Figure 3D). These results demonstrate the requirement of lower dosages for longer GAA A-GOs for optimal *FXN* mRNA upregulation when delivered by gymnosis. Of note, we did not observe significant upregulation of *FXN* using GAA_{16}^{High} and GAA_{19}^{High} (Figures 4A and 4B).

The CTT ONs were used as control since it was established in the transfection experiments that these resulted in the opposite effect, thereby reducing *FXN* mRNA levels. The silencing effect of CTT_{15}, CTT_{16}^{High}, CTT_{19}^{High}, and CTT_{24} in GM03816 showed a dose-response for the concentration interval 0.18 to 6 μM, with significantly greater *FXN* downregulation at higher concentrations (Figures 3A, 3C, 4A, and 4B). In 4869 fibroblasts, the concentrations tested of 0.37 and 0.75 μM also resulted in a significant downregulation of *FXN* expression (Figure 3D).

Additionally, we evaluated the effect of GAA_{24} and CTT_{24} on the mRNA of other repeats containing genes in 4869 fibroblasts after 4-day-long $CaCl_2$-assisted gymnosis. To this end, we selected five genes with varying repeat length, location, and orientation (Figure S7). As determined by RT-qPCR, GAA_{24} upregulated gene expression when the repeats are in the 3′UTR regardless of their orientation. Treatment with CTT_{24} resulted in gene downregulation. From these results, it is clear that both ONs affect other repeat containing genes; however, additional studies are needed to comprehensively determine the biological consequences of off-target binding.

To rule out the possibility that the *FXN* activation observed is an off-target effect, we treated an unaffected cell line (6718) that contains approximately 6/6 GAA•TTC repeats. Similar to FRDA-treated fibroblasts, 6718 fibroblasts were gymnotically treated with 24-mers with selected concentrations of ONs in medium supplemented with 9 mM $CaCl_2$. The cells were harvested 4 days post-treatment

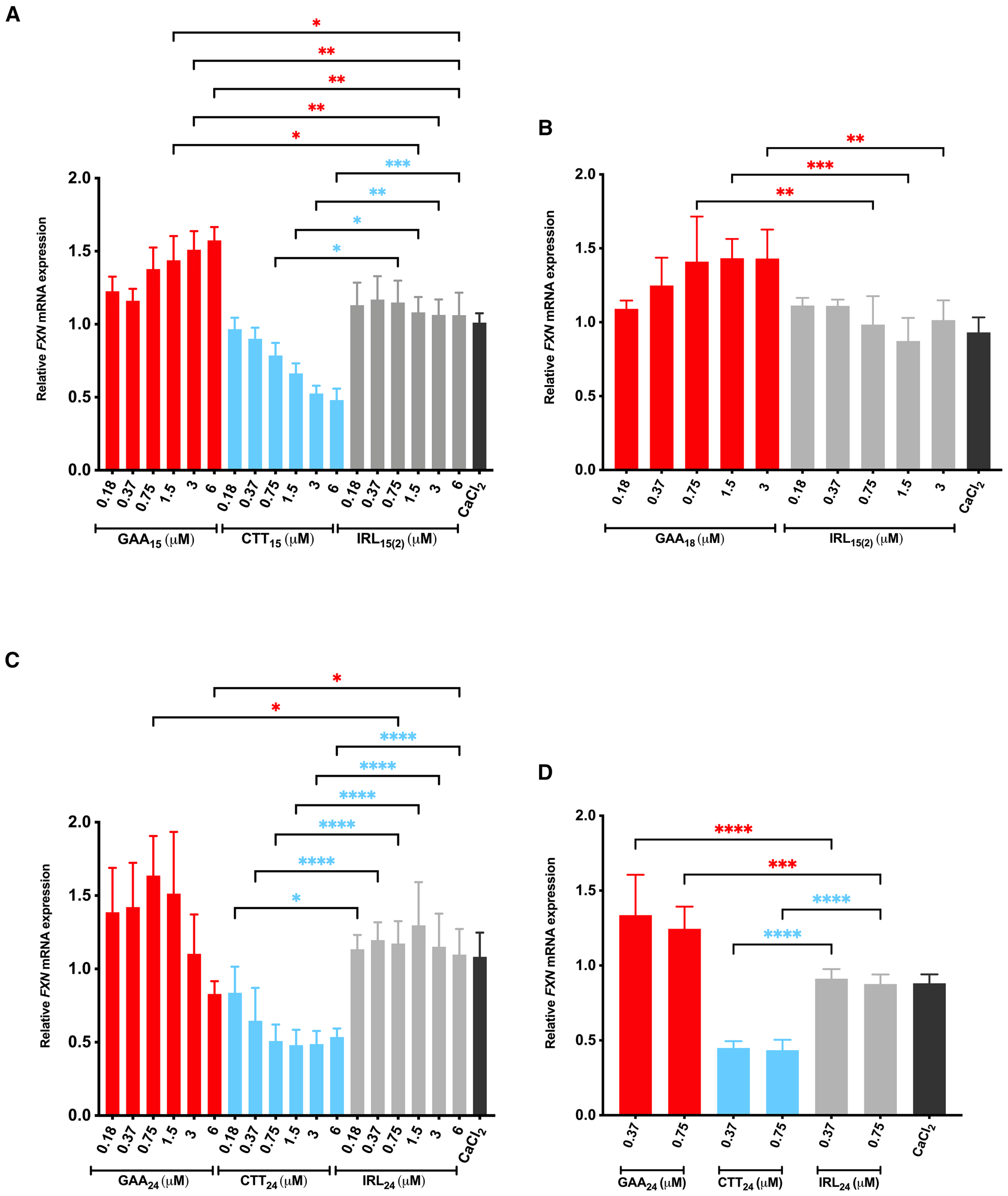

Figure 3. Dose-dependent *FXN* mRNA expression after gymnotic delivery of ONs

Female FRDA patient-derived fibroblasts carrying 330/380 GAA•TTC repeats (GM03816) (A–C) were treated with ONs at concentrations ranging from 0.18 to 6 μM and (D) 4869 fibroblasts were treated with selected concentrations of 0.37 and 0.75 μM, both in a medium supplemented with 9 mM $CaCl_2$. Four days post-treatment the cells were harvested, and *FXN* mRNA levels were analyzed. The values were normalized to *HPRT1* as a reference gene and compared with NT cells. Results are presented as mean ± SD ($n \geq 3$). Statistics for sections (A), (B), and (D) were performed with one-way ANOVA Multiple Comparison (Šidák) toward the corresponding concentration of IRL ON (*$p < 0.05$, **$p < 0.01$). Statistical analysis for (C) was performed using the Kruskal-Wallis test. IRL ONs are randomly 15- or 24-nt-long ONs that are not complementary to any related genes in this project.

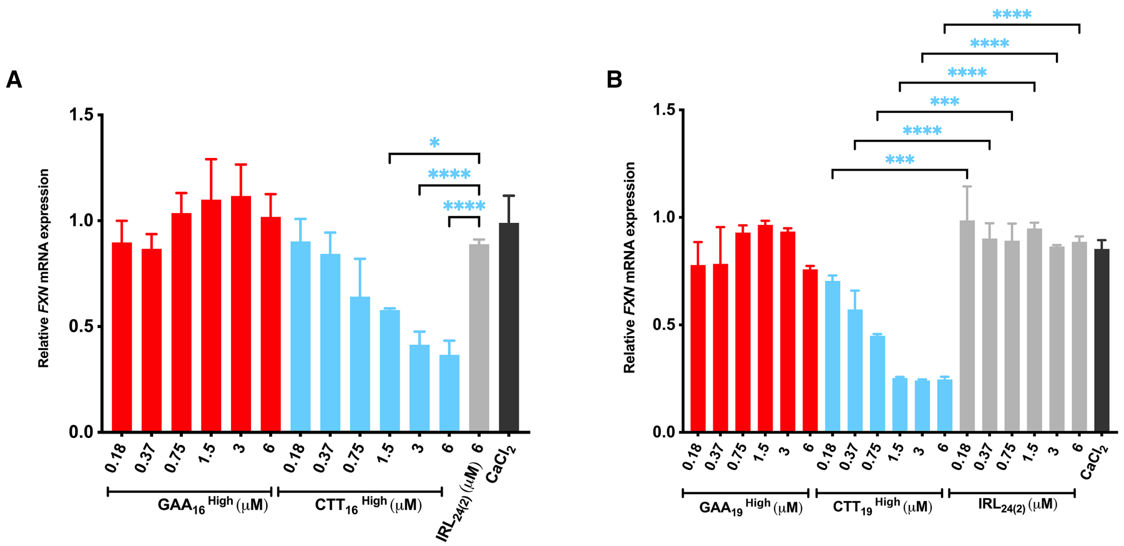

Figure 4. Dose-response of gymnotically-delivered ONs on the *FXN* mRNA expression

GM03816 cells were treated with (A) 16-mer and (B) 19-mer ONs at concentrations ranging from 0.18 to 6 μM in medium supplemented with 9 mM $CaCl_2$. After 4 days of treatment, the cells were harvested, and *FXN* mRNA expression levels were analyzed. The values were normalized to *HPRT1* levels as the reference gene and were compared to NT cells. Results are presented as mean ± SD. Statistics were performed with one-way ANOVA multiple comparisons (Šidák), toward the IRL control (**$p < 0.01$, ***$p < 0.001$, ****$p < 0.0001$).

and FXN mRNA and protein levels were determined using RT-qPCR and western blot, respectively. Neither GAA_{24} resulted in FXN mRNA and protein upregulation, nor CTT_{24} resulted in FXN mRNA and protein downregulation, which suggests that the observed effect is dependent on the presence of repeat expansion (Figure S3).

Furthermore, selected ONs with 40% LNA content did not affect cell viability, as determined by the WST-1 assay across all tested concentrations (Figure S4). Based on these results, increasing the LNA content of GAA A-GOs reduces their activity above a certain threshold of 3–6 μM in a gymnotic context. Moreover, the longer the GAA A-GOs, the more effective they were at lower doses.

GAA A-GOs upregulate FXN protein expression

Following the evaluation of the optimal length and sequence of GAA A-GOs in *FXN* mRNA upregulation by RT-qPCR, we aimed to assess if that effect can be translated to protein production. FRDA fibroblasts (GM03816) were transfected with 100 nM of ONs with 40% LNA content (GAA_{15}, GAA_{18}, GAA_{24}, CTT_{15}, CTT_{24}, $IRL_{15(1)}$, $IRL_{15(2)}$, and $IRL_{24(1)}$). The cells were harvested after 4 days, and protein expression was analyzed by western blott. Similar to the mRNA experiments, both CTT_{15} and CTT_{24} downregulated the FXN protein expression compared with cells treated with the control ONs (IRL_{24} and IRL_{15}) (Figure 5A). GAA_{24} treatment showed the desired outcome of a significant increase in the FXN protein levels, which relates to the increased levels of *FXN* mRNA. Next, we examined gymnotic delivery conditions using the 24-nucleotide-long GAA A-GO. GAA_{24} and the corresponding control ONs, $IRL_{15(2)}$, and CTT_{24} were gymnotically delivered to GM03816 cells at concentrations ranging from 0.37 to 1.5 μM. Our data confirmed that the GAA_{24} A-GO significantly upregulated FXN protein expression under these conditions (Figures 5C and 5D). Moreover, cell treatment with the CTT_{24} reduced FXN protein levels (Figures 5C and 5D), following the previous trend where increased concentrations were more efficient (0.37 μM and 1.5 μM, respectively), in line with the results obtained by analysis of mRNA expression levels.

GAA A-GOs upregulate *FXN* mRNA expression in different FRDA-patient-based cell models

Most FRDA patients carry 600–900 GAA•TTC repeat expansions in the *FXN* gene,[3] with a maximum reported of 1,700 repeats.[46] After validating the efficiency of GAA A-GOs in enhancing *FXN* mRNA expression in FRDA cell models carrying 330/380 and 294/405 GAA•TTC repeats, we hypothesized that A-GOs should similarly function in cell models with a higher number of GAA•TTC repeats. For that purpose, female FRDA patient-derived fibroblasts carrying approximately 780/1410 GAA•TTC repeats (GM03665) were transfected with 200 nM of GAA and CTT ONs. To assess how the length alters ON potency in these cell lines, different lengths of GAA A-GOs were tested along with the selected cognates CTT_{15} and CTT_{24}, and corresponding controls IRL_{15} and IRL_{24}. Regarding LNA content, in the low GAA•TTC repeat fibroblasts (GM03618), the best-performing ON design included 38%–40% LNA. Based on this, GM03665 fibroblasts were treated with ONs containing 38%–40% LNA content and varying lengths. As a control, nontreated cells were also

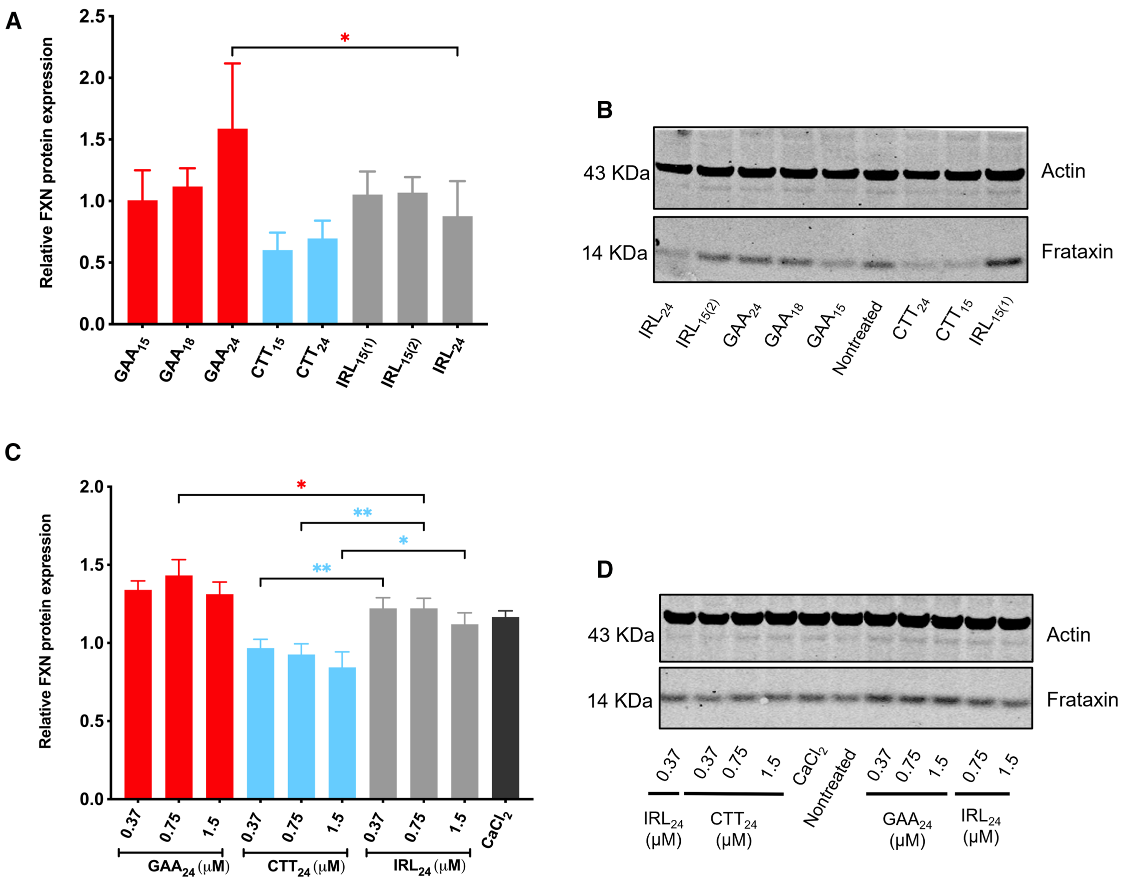

Figure 5. GAA A-GOs increase the FXN protein levels

(A) Increased effect of ONs with 40% of LNA on FXN protein expression 4 days post-transfection with 100 nM ONs in GM03816 cells using Lipofectamine LTX. The FXN protein was normalized to Actin levels as a reference gene and relative FXN expression is shown after normalizing to the control NT cells. Results are presented as mean ± SD ($n \geq 3$). (B) Representative western blot of the treatment presented in (A). (C) The FXN protein levels were measured by western blotting 4 days after the gymnotic delivery of GAA_{24}, CTT_{24}, and $IRL_{15(2)}$ in the presence of a medium supplemented with 9 mM $CaCl_2$. IRL ON ($IRL_{(15)2}$) is random 15 nt long ON that is not complementary to any related genes in this project and the LNA composition is the same as the GAA and CTT ONs. The values were normalized to Actin levels as a reference gene and compared with NT cells. Results are presented as mean ± SD ($n = 3$). Statistics were performed with one-way ANOVA multiple comparisons (A: Šidák and B: Fisher's least significant difference test), toward corresponding controls. (D) Representative western blot of the treatment presented with GAA_{24}, CTT_{24}, and $IRL_{15(2)}$ in medium supplemented with 9 mM $CaCl_2$ collected 4 days after treatments.

included. Cells were harvested 4 days after transfection, and *FXN* mRNA levels were assessed by RT-qPCR (Figure 6). A similar experiment was performed at a concentration of 100 nM (Figure S6).

The results show that *FXN* mRNA levels are significantly upregulated when GM03665 fibroblasts were transfected with GAA_{15} and GAA_{24} at 200 nM (Figure 6). Similar to what was observed in GM03816 fibroblasts, the obtained *FXN* upregulation reached a maximum effect of 1.6–1.7-fold increase. Again, the upregulation correlated positively with the ON length, as increasing GAA A-GO length significantly increased *FXN* expression. The CTT_{15} and CTT_{24} ON resulted, as expected, in significant downregulation of *FXN* mRNA (Figure 6). These data validate the efficiency of A-GOs in the context of different FRDA patient-derived fibroblasts containing a higher number of expanded GAA•TTC repeats.

Gymnotic delivery of GAA_{24} A-GOs successfully increases *FXN* mRNA expression in the higher-repeats cell line

In the lower number of repeat GM03618 fibroblasts, the best-performing combination regarding ON length and LNA content was the 24-mer GAA A-GO containing 38%–40% LNA content. Similarly, GM03665 fibroblasts transfected with GAA_{24} containing the same LNA amount resulted in the highest *FXN* upregulation. Based on this, GM03665 fibroblasts were treated with GAA_{24} containing 38%–40% LNA in medium enriched with 9 mM $CaCl_2$, at final concentrations ranging from 0.18 to 3 μM. A similar treatment was carried out using the corresponding CTT_{24}. For the IRL_{24}, only selected concentrations were used. Nontreated cells were also included. Cells were harvested 4 days after treatments and *FXN* mRNA levels were assessed by RT-qPCR. Cell treatment with GAA_{24} showed a dose-response, and the A-GO significantly upregulated *FXN* mRNA levels

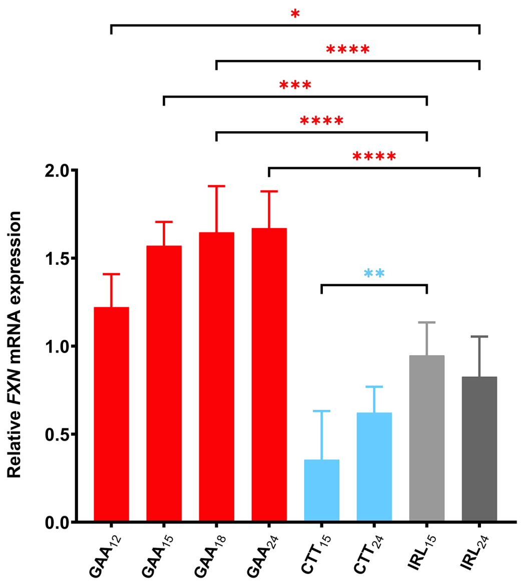

Figure 6. Increasing the GAA A-GO length enhances *FXN* upregulation in a FRDA cell model with a higher number of GAA·TTC repeats

GM03665 fibroblasts were treated with ONs of different lengths of GAA, CTT, and IRL at 200 nM. Treated and nontreated cells were harvested 4 days after transfection, and *FXN* mRNA levels were assessed by RT-qPCR. The values were normalized to *HPRT1* and were compared with NT cells. Results are presented as mean ± SD ($n \geq 3$). Statistics were performed with one-way ANOVA Multiple Comparison (Šidák), toward control ONs (*$p < 0.05$, **$p < 0.01$, ***$p < 0.001$).

at 0.37 μM and 0.75 μM, with a maximum effect of a 1.6-fold increase (Figure 7). This pattern is comparable to what was observed in GM03816 and 4869 fibroblasts, where lower concentrations of GAA_{24} led to optimal *FXN* mRNA upregulation (Figures 3C and 3D). As expected, the CTT_{24} induced significant downregulation of *FXN* mRNA levels in all tested concentrations, in a dose-response manner (Figure 7). Hence, CTT_{24} behaved here again in an opposite manner compared with GAA_{24}, being significantly more potent at higher concentrations (3 μM) compared with lower concentrations (0.18 μM). This is in accordance with our findings observed in GM03816 fibroblasts. Altogether, these data confirm the efficiency of gymnotically delivered GAA A-GOs to increase *FXN* expression in different FRDA cell models.

DISCUSSION

In this study, we designed modified, single-strand A-GOs to target a non-B-DNA structure formed at expanded GAA•TTC repeats at intron 1 of the *FXN* gene, as a potential therapeutic approach for FRDA. We designed several PS-modified LNA/DNA mixmer ON with varying lengths, LNA content, and LNA positioning. The GAA A-GOs, which are complementary to the template strand, significantly upregulated FXN mRNA and protein expression in a dose-dependent way. In contrast, the CTT ONs are complementary to the coding strand and significantly reduced FXN mRNA and protein levels. We assessed their efficiency in three FRDA-patient-derived (two females and one male) cell models containing different GAA•TTC repeat numbers. Using both lipid-based transfection and gymnosis-delivery, the GAA A-GOs led to a significant increase in FXN mRNA and protein expression. However, this was only observed when a specific number of LNA modifications at certain positions within the sequence were used, highlighting that the design of the GAA A-GO is crucial for its efficacy in DNA double-strand invasion. In contrast to the ability of GAA A-GOs to increase *FXN* mRNA expression, CTT ONs consistently reduced FXN mRNA and protein expression.

In FRDA, the expanded GAA•TTC repeats lead to the formation of non-canonical parallel or antiparallel triplex rather than the common, intrinsic B-DNA structure.[28] The parallel triplex is formed when the CTT strand of the duplex folds back and forms hydrogen bonds with the undisrupted part of the duplex, leaving the GAA single-stranded.[47] The formation of an intracellular triplex leads to RNA polymerase II blockage and, eventually, causing transcriptional silencing of *FXN* mRNA expression.[48]

As the pathogenic, expanded GAA•TTC repeats are located in an intronic region of the *FXN* gene, the use of sequence-specific A-GOs targeting chromosomal DNA has great potential. The GAA A-GOs are expected to bind to the expanded repeat region and abolish the formation of the H-DNA structure. This has previously been validated based on chemical and structural probing of the DNA complexes formed in the presence of modified ONs.[39] We hypothesized that the prevention of H-DNA formation, or any other possible non-B-DNA structure, can potentially lead to enhanced transcription, elongation and upregulation of *FXN* gene expression. CTT ONs with the same design and length as the GAA A-GOs were also explored. CTT ONs are believed to bind sequence-specifically to the formed single strand $(GAA)_n$ at the *FXN* gene, making the H-DNA more stable. Additionally, they can bind the repeat region and sterically block the pre-mRNA.[48] Furthermore, we recently showed that both our ONs reduce GAA•TTC expansion frequency in an experimental model system.[41] Here, we further optimized these ONs and assessed their potency in *FXN* upregulation, a key step in the development of FRDA therapeutics.

In contrast to the conventional approach of ASOs, A-GOs are designed to specifically target and modulate gene expression at the chromosomal level. Targeting and disrupting an intramolecular triplex is complex, especially in a region that is believed to be epigenetically inactivated.[48] We therefore evaluated the effect of GAA A-GOs on *FXN* levels by varying their lengths and LNA content. Increasing the LNA proportion had no beneficial effect on *FXN* mRNA upregulation. This could be explained by the need for a balance between GAA A-GO invasion of genomic DNA with subsequent binding and disruption of the triplex, followed by A-GO disassociation from DNA, thereby enabling the RNA polymerase to elongate. This suggests that although LNA-modified ONs show greater affinity and stability compared with unmodified ONs,[49] their sequence composition also needs to be well adjusted. We found that increasing the length of

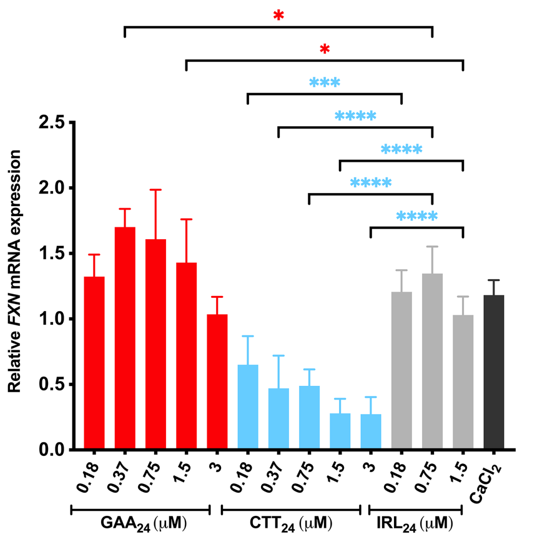

Figure 7. Dose-dependent effect of gymnotically-delivered GAA_{24} on *FXN* expression

FRDA fibroblasts containing a higher number of GAA•TTC repeats (GM03665) were treated with GAA_{24} and CTT_{24} ONs at concentrations ranging from 0.18 to 3 μM in medium supplemented with 9 mM $CaCl_2$. For IRL_{24}, only selected concentrations were used. Treated and NT cells were harvested 4 days post-treatments, and *FXN* mRNA levels were assessed by RT-qPCR. The values were normalized to *HPRT1* levels as reference gene and were compared with NT cells. Results are presented as mean ± SD ($n \geq 3$). Statistics were performed with one-way ANOVA multiple comparisons (Holm- Šidák) toward control ONs (*$p < 0.05$, **$p < 0.01$, ***$p < 0.001$, ****$p < 0.0001$).

GAA A-GOs with an optimized LNA content enhanced *FXN* mRNA upregulation in a dose-dependent fashion. Thus, GAA_{24} holds a better promise for *FXN* upregulation at lower concentrations, rendering it of interest as a potential therapeutic agent for the treatment of FRDA. Having examined this in three cell lines originating from patients carrying different full-penetrance allele sizes, we observed that the maximum upregulation was reached at the same concentration range in all three cell lines (GM03816, 4869, and GM03665). These results could indicate the same optimal dosage for FRDA patients independently of their allele sizes. The upregulation we achieved is physiologically relevant, as in FRDA heterozygous carriers express 50% less FXN while developing no disease phenotype.[15,50] Furthermore, the level of FXN upregulation needs to be well adjusted as it has been shown that overexpression of FXN causes toxicity.[51] The overexpression of human FXN was toxic not only to the heart, a key target organ in FRDA, but also to the liver.[51] Importantly, the A-GOs used in this study seem to be GAA•TTC repeats-dependent as the treatment of healthy fibroblasts did not alter either FXN mRNA or protein expression. Furthermore, a significant *FXN* mRNA upregulation was achieved by shorter A-GOs (e.g., GAA_{15}), which is also of interest in a treatment context, since *in vivo* uptake of A-GOs may be length dependent. The results using GAA A-GOs are therefore in agreement with our hypothesis that the balance between the capacity of GAA A-GOs for DNA invasion/disruption of the triplex and subsequent dissociation from genomic DNA needs careful optimization with respect to design and dosage.

Moreover, in contrast to a mechanism whereby the H-DNA is dissolved by GAA A-GOs, for CTT ONs an effect on DNA as well as on pre-mRNA is likely. Irrespective of the mechanism of action, CTT ONs with enhanced hybridization properties significantly reduced the *FXN* mRNA expression regardless of LNA content and its position. This was shown in all tested FRDA patient cell lines. In alignment with the *in vivo* results presented by Kilikevicius et al., our *in vitro* findings in FRDA patient-derived cell lines confirm that the same ASO sequence (CTT) utilized in their animal model leads to downregulation of *FXN* expression in our cell models as well.[38]

It is worth mentioning that A-GOs require higher concentrations to target chromosomal DNA than ASOs targeting pre-mRNA or mRNA. This is counterintuitive, as there are only two binding regions in the *FXN* gene compared with constantly produced RNA targets. However, chromosomal DNA is tightly packed into chromatin structures, limiting accessibility and requiring more A-GOs to penetrate and bind to specific genomic regions effectively.[52] Additionally, A-GOs must compete with natural DNA- and RNA-binding proteins that also interact with these sequences, necessitating a higher dosage to achieve sufficient binding.[53] In contrast, ASOs target single-stranded RNA, which is more accessible, reducing the required concentration for effective inhibition or modulation. This is why higher concentrations of A-GOs ∼100 nM during transfection are needed to observe increases, compared with the low nanomolar concentrations previously reported for the same cell model.[36,37]

We have recently reported that CAG•CTG repeat-targeting A-GOs had a distinct effect on the *HTT* locus, whose expanded repeats lead to Huntington's disease (HD). Expansion of CAG•CTG repeats results in a toxic gain of function and hence the aim of the A-GOs targeting these repeats is to reduce the levels of HTT mRNA and protein. This has been shown in HD patient-derived fibroblasts and neural stem cells differentiated from induced pluripotent stem cells.[54,55] The underlying mechanism of experimental A-GO therapy differs between FRDA and HD, thereby, a different design regarding the corresponding A-GOs is necessary. Moreover, the non-B-DNA structure of the affected genes is not the same in FRDA and HD. In the *HTT* locus, the CAG repeats are likely to adopt hairpin conformation, whereas in the *FXN* gene, a triplex/H-DNA is formed.[56] For the *HTT* locus, a high LNA content was essential for obtaining efficient DNA binding and an inhibitory effect on *HTT* expression.[44] Unlike our findings in FRDA, A-GOs containing 60% LNA were highly efficient in HD patient-derived cell lines.

Our findings also indicate that GAA_{24} upregulated genes when repeats are in the 3′ UTR, while CTT_{24} consistently downregulated gene expression, highlighting potential off-target effects. However, further *in vivo* studies are needed to assess these effects in a physiological context. Given the severity of FRDA, minor off-target effects may be acceptable if therapeutic benefits outweigh the risks. Future

work should focus on optimizing A-GO design to enhance specificity while maintaining efficacy.

While we examined several different A-GO constructs, compared with the extensive testing of compounds generally performed in the pharmaceutical industry, our ON catalog has been modest in size. Continued optimization of these A-GOs may therefore yield even more efficient lead compounds to be developed as therapeutics. Nevertheless, we demonstrated that the A-GO targeting concept is valid in three FRDA male and female patient-derived cell lines carrying different GAA•TTC repeat-expanded alleles. The A-GOs that have been previously reported to disrupt H-DNA formation now show for the first time the capacity to significantly upregulate *FXN* expression. This suggests that they can potentially be developed as a treatment option for FRDA as well as tools for investigating the dynamic DNA structures at the *FXN* locus.

MATERIAL AND METHODS

Oligonucleotides

LNA/DNA mixmers were purchased from Eurogentec S.A. (Seraing, Belgium) or were synthesized at the Nucleic Acid Center, University of Southern Denmark. The ONs were purified by reversed-phase HPLC, and their composition was confirmed by MALDI-TOF mass spectrometry. The GAA A-GOs were designed to target the pyrimidine motif triplex formed at the *FXN* intron expanded GAA•TTC repeats while the CTT ONs were designed to bind to the single-stranded GAA region of the triplex or to the pre-mRNA. Control ONs were designed with the same LNA composition and percentage. The control ONs (irrelevant [IRL]) were random ONs with corresponding length and LNA content, which are not complementary to any related genes in this project. The complete list of ONs used in this study is presented in Table 1.

Cell culture, transfection, and gymnotic delivery

The female primary fibroblasts GM03816 and GM03665 derived from FRDA patients were obtained from the Coriell Institute (Camden, NJ). The male 4869 FRDA fibroblasts and the 6718 control fibroblasts were obtained from the Friedreich's Ataxia Cell Line Repository (FACLR). The 6718, GM03816, 4869, and the GM03665 cells contain approximately 6/6 GAA•TTC repeats, 330/380 GAA•TTC repeats, 294/405 GAA•TTC repeats and 780/1410 GAA•TTC repeats, respectively. Fibroblasts were maintained in a humidified incubator at 37°C with 5% CO_2. Cells were grown in Dulbecco's Modified Eagle's Medium (DMEM) with pyruvate and low glucose (Gibco, Fisher Scientific, Hampton, NH), supplemented with 15% Fetal Bovine Serum (FBS) (Gibco, Thermo Fisher Scientific, Waltham, MA). Lipofectamine LTX with PLUS reagent (Invitrogen by Thermo Fisher Scientific, Waltham, MA) was used to transfect the ONs according to the manufacturer's recommended protocol. Briefly, cells were seeded a day before transfection at 1×10^4, 8×10^4, or 3×10^5 cells per well in 96-, 24-, or 6-well plates, respectively. ONs were formulated with Lipofectamine LTX and PLUS reagent (Invitrogen by Thermo Fisher Scientific, Waltham, MA) at a final concentration of 100 or 200 nM in OptiMEM reduced serum medium (Gibco, Fisher Scientific, Hampton, NH). For gymnotic delivery, ONs were added freshly to the medium supplemented with 9 mM $CaCl_2$[47] for 4 days.

DNA isolation

Genomic DNA from fibroblasts was isolated with a DNeasy Blood & Tissue kit (QIAGEN, Hilden, Germany) according to the manufacturer-recommended protocol. Genomic DNA concentration and purity were determined with a NanoPhotometer (Implen, München, Germany).

PCR amplification

Amplification of GAA•TTC repeats at the *FXN* locus was performed as previously described with the following forward (Fw) and reverse (Rv) primers: GAA-fw: GGCTTGAACTTCCCACACGTGTT and rv: AGGACCATCATGGCCACACTT in HotStarTaq Plus Master Mix Kit (QIAGEN, Hilden, Germany).[18] PCR reaction was performed in 20 μL final volume containing 50 ng of genomic DNA. The PCR program was set to 3 min of denaturation at 94°C, which continued with 20 cycles of 20 s of denaturation at 94°C, 30 s of annealing at 64°C, and 5 min of elongation at 68°C, followed by nine cycles of 20 s of denaturation at 94°C and 5 min of elongation at 68°C, with each subsequent elongation step increased by 15 s. Finally, a 7-min extension at 68°C was performed. The PCR products were analyzed with 0.7% agarose gels with SYBR Gold Nucleic Acid Gel Stain (Invitrogen by Thermo Fisher Scientific, Waltham, MA).

RNA isolation and reverse-transcription quantitative PCR

Total RNA was isolated with Tri-reagent (Sigma-Aldrich/Merck, St. Louis, MO) or RNeasy plus kit (QIAGEN, Hilden, Germany) according to the manufacturer's protocol. The quantity and quality of RNA were measured with NanoPhotometer (Implen, München, Germany). 200 ng of total RNA was used for cDNA synthesis with the High-Capacity cDNA Reverse Transcription Kit using random primers (Applied Biosystems, Waltham, MA). RT-qPCR was performed by the CFX96 or CFX Opus Real-Time PCR system (Bio-Rad, Hercules, CA) using TaqMan Fast Advanced Master Mix (Applied Biosystems, Waltham, MA) with 20 ng of cDNA as a template. *FXN* Exon4-Exon5 was amplified using the primers and probe from Zanella et al.[57] Normalization was performed using hypoxanthine phosphoribosyltransferase 1 (*HPRT1*) as a housekeeping gene. For the off-target gene analysis, we used 8 ng (*ADK*, *KAT6B*, *RIN2*) or 40 ng (*PRDM10*, *RPS6KA5*) of cDNA. The sequences of all primer and probe sets are presented in Table S1. The data were analyzed with CFX Maestro software (Bio-Rad, Hercules, CA) using the ΔΔCq method. Moreover, the Cq values of the *HPRT1* housekeeping gene remained consistent across experiments, ensuring reliable normalization for gene expression analysis. Selected *HPRT1* Cq values are displayed in Figure S5.

Western blotting

Cells from 6-well plates were trypsinized (Gibco, Fisher Scientific, Hampton, NH) and collected in Eppendorf tubes. Cells were lysed with RIPA buffer for 30 min on ice following centrifugation at top speed for 15 min at 4°C. 10x NuPAGE Sample Reducing Agent

(Invitrogen, Thermo Fisher Scientific, Waltham, MA) and 4x NuPAGE LDS Sample Buffer (Invitrogen, Thermo Fisher Scientific, Waltham, MA) were added to the supernatant after centrifugation. The samples were heated at 75°C for 10 min before loading on the gel. Proteins were separated on NuPAGE 4% to 12%, Bis-Tris gels (Invitrogen, Thermo Fisher Scientific, Waltham, MA) at 70 V for 20 min following 90 min at 130 V. Gels were transferred using iBlot 2 Transfer Stacks, nitrocellulose (Invitrogen, Thermo Fisher Scientific, Waltham, MA) and iBlot2 Gel Transfer Device (Invitrogen, Thermo Fisher Scientific, Waltham, MA). The membranes were blocked with Odyssey TBS Blocking Buffer (LI-COR Biosciences, Lincoln, NE) for 1 h. Blocked membranes were probed with anti-FXN primary antibody (ab110328, Abcam, Cambridge, UK) (1:500) and anti-Actin (1:10^6) (A1978, Sigma-Aldrich/Merck, St. Louis, MO) as a reference. The primary antibodies were diluted in a 1:1 ratio of Phosphate-buffered saline with 0.1% Tween 20 (PBST) and blocking buffer and incubated at 4°C on a rocking plate shaker overnight. After primary antibody incubation, the membranes were washed five times for 5 min at room temperature with 1x PBST and then incubated with a secondary antibody IRDye 800Cw goat anti-mouse immunoglobulin G (1:40,000) (LI-COR Biosciences, Lincoln, NE) for 1 h at room temperature. Membranes were washed five times for 5 min at room temperature with 1x PBST and 2 × 3 min with PBS before scanning. The signals were detected with an Odyssey imager (LI-COR Biosciences, Lincoln, NE) at 800 nm.

Viability assay

To assess the viability of the cells upon ON treatments, the WST-1 assay (Merck, St. Louis, MO) was used according to the manufacturer's recommended protocol. Briefly, cells were cultured in a 96-well plate. The day after seeding, the cells were treated with the ONs as stated above. Two days after treatment, media was substituted with fresh media containing 10 μL (1:10 dilution) of WST-1 reagent and incubated at 37°C with 5% CO_2 for 2 h. The signals were measured with SpectraMax i3x (Molecular Devices, San Jose, CA) at 450 nm with 600 nm as reference wavelength. The relative value of cell viability was calculated based on the ratio of treated and nontreated cells at 450 nm wavelength.

Statistical analysis

To ensure the appropriate statistical tests were applied, the data were first assessed for normality using the Shapiro-Wilk test. If the data followed a normal distribution, one-way ANOVA, Šidák, Dunnet, and Holm-Šidák multiple comparisons tests were performed to determine if there were statistically significant differences between the groups. When no correction were applied, Fisher's LSD test was used. In cases where the data did not meet the normality assumption, non-parametric tests, specifically the Kruskal-Wallis and Dunn's tests, were employed to analyze the differences between the groups. For viability experiments, two-way ANOVA and Turkey multiple comparison tests were employed. This approach ensured that the most suitable statistical methods were used based on the distributional characteristics of the data.

DATA AVAILABILITY

The data underlying this article will be shared on reasonable request by the corresponding author.

ACKNOWLEDGMENTS

Funding was kindly provided from Hjärnfonden [FO 2022-0257] (C.I.E.S.), The Swedish Research Council, Swelife-Vinnova, CIMED (Center for Innovative Medicine) and Region Stockholm (O.S., N.M., C.I.E.S., and R.Z.). This project has also received funding from the European Union's Horizon 2020 Research and Innovation Programme under grant agreement no. 956070 (S.M.; granted to C.I.E.S.) and the Novo Nordisk Foundation [NNF21OC0072778] "Pioneer Innovator 2-2021" (N.M., T.U.). The cells obtained from the Friedreich's Ataxia Cell Line Repository (FACLR) were kindly provided by M. N. and J.S.N. M.N. is supported by the Congressionally Directed Medical Research Programs (CDMRP) under Award Number HT9425-23-1-0337 and J.S.N. is supported by Friedreich's Ataxia Research Alliance.

AUTHOR CONTRIBUTIONS

All authors declare a contribution to this paper. R.Z., C.I.E.S., and N.M. designed and planned the study with input from P.B., P.T.J., and J.W. N.M., S.M., T.U., C.M.V., F. F., and O.S. performed and analyzed experiments. J.W. and P.T.J. contributed to chemical synthesis. N.M. wrote the first draft of the manuscript. S.M., T.U., C.S.J.R., P.B., P.T. J., J.W., J.S.N., M.N., C.I.E.S., and R.Z. took part in the revision of the manuscript for important intellectual content. All authors reviewed and approved the final version of the manuscript.

DECLARATION OF INTERESTS

R.Z. has a granted patent for diagnosis and treatment of Friedreich's ataxia.

SUPPLEMENTAL INFORMATION

Supplemental information can be found online at https://doi.org/10.1016/j.omtn.2025.102541.

REFERENCES

1. Koeppen, A.H. (2011). Friedreich's ataxia: pathology, pathogenesis, and molecular genetics. J. Neurol. Sci. *303*, 1–12.
2. Ocana-Santero, G., Díaz-Nido, J., and Herranz-Martín, S. (2021). Future Prospects of Gene Therapy for Friedreich's Ataxia. Int. J. Mol. Sci. *22*, 1815.
3. Pandolfo, M. (2009). Friedreich ataxia: the clinical picture. J. Neurol. *256*, 3–8.
4. De Castro, M., García-Planells, J., Monrós, E., Cañizares, J., Vázquez-Manrique, R., Vílchez, J.J., Urtasun, M., Lucas, M., Navarro, G., Izquierdo, G., et al. (2000). Genotype and phenotype analysis of Friedreich's ataxia compound heterozygous patients. Hum. Genet. *106*, 86–92.
5. Campuzano, V., Montermini, L., Moltò, M.D., Pianese, L., Cossée, M., Cavalcanti, F., Monros, E., Rodius, F., Duclos, F., Monticelli, A., et al. (1996). Friedreich's ataxia: autosomal recessive disease caused by an intronic GAA triplet repeat expansion. Science (New York, NY) *271*, 1423–1427.
6. Cossée, M., Dürr, A., Schmitt, M., Dahl, N., Trouillas, P., Allinson, P., Kostrzewa, M., Nivelon-Chevallier, A., Gustavson, K.H., Kohlschütter, A., et al. (1999). Friedreich's ataxia: point mutations and clinical presentation of compound heterozygotes. Ann. Neurol. *45*, 200–206.
7. Delatycki, M.B., and Bidichandani, S.I. (2019). Friedreich ataxia- pathogenesis and implications for therapies. Neurobiol. Dis. *132*, 104606.
8. Campuzano, V., Montermini, L., Lutz, Y., Cova, L., Hindelang, C., Jiralerspong, S., Trottier, Y., Kish, S.J., Faucheux, B., Trouillas, P., et al. (1997). Frataxin is reduced in Friedreich ataxia patients and is associated with mitochondrial membranes. Hum. Mol. Genet. *6*, 1771–1780.
9. Das, D., Patra, S., Bridwell-Rabb, J., and Barondeau, D.P. (2019). Mechanism of frataxin "bypass" in human iron-sulfur cluster biosynthesis with implications for Friedreich's ataxia. J. Biol. Chem. *294*, 9276–9284.
10. Pandey, A., Gordon, D.M., Pain, J., Stemmler, T.L., Dancis, A., and Pain, D. (2013). Frataxin directly stimulates mitochondrial cysteine desulfurase by exposing

substrate-binding sites, and a mutant Fe-S cluster scaffold protein with frataxin-bypassing ability acts similarly. J. Biol. Chem. *288*, 36773–36786.

11. Tai, G., Corben, L.A., Yiu, E.M., Milne, S.C., and Delatycki, M.B. (2018). Progress in the treatment of Friedreich ataxia. Neurol. Neurochir. Pol. *52*, 129–139.

12. Evans-Galea, M.V., Pébay, A., Dottori, M., Corben, L.A., Ong, S.H., Lockhart, P.J., and Delatycki, M.B. (2014). Cell and gene therapy for Friedreich ataxia: progress to date. Hum. Gene Ther. *25*, 684–693.

13. Ohshima, K., Montermini, L., Wells, R.D., and Pandolfo, M. (1998). Inhibitory effects of expanded GAA.TTC triplet repeats from intron I of the Friedreich ataxia gene on transcription and replication in vivo. J. Biol. Chem. *273*, 14588–14595.

14. Burnett, R., Melander, C., Puckett, J.W., Son, L.S., Wells, R.D., Dervan, P.B., and Gottesfeld, J.M. (2006). DNA sequence-specific polyamides alleviate transcription inhibition associated with long GAA.TTC repeats in Friedreich's ataxia. Proc. Natl. Acad. Sci. USA *103*, 11497–11502.

15. Bidichandani, S.I., Ashizawa, T., and Patel, P.I. (1997). Atypical Friedreich ataxia caused by compound heterozygosity for a novel missense mutation and the GAA triplet-repeat expansion. Am. J. Hum. Genet. *60*, 1251–1256.

16. Bartolo, C., Mendell, J.R., and Prior, T.W. (1998). Identification of a missense mutation in a Friedreich's ataxia patient: implications for diagnosis and carrier studies. Am. J. Med. Genet. *79*, 396–399.

17. De Biase, I., Rasmussen, A., Endres, D., Al-Mahdawi, S., Monticelli, A., Cocozza, S., Pook, M., and Bidichandani, S.I. (2007). Progressive GAA expansions in dorsal root ganglia of Friedreich's ataxia patients. Ann. Neurol. *61*, 55–60.

18. Long, A., Napierala, J.S., Polak, U., Hauser, L., Koeppen, A.H., Lynch, D.R., and Napierala, M. (2017). Somatic instability of the expanded GAA repeats in Friedreich's ataxia. PLoS One *12*, e0189990.

19. De Biase, I., Rasmussen, A., Monticelli, A., Al-Mahdawi, S., Pook, M., Cocozza, S., and Bidichandani, S.I. (2007). Somatic instability of the expanded GAA triplet-repeat sequence in Friedreich ataxia progresses throughout life. Genomics *90*, 1–5.

20. Punga, T., and Bühler, M. (2010). Long intronic GAA repeats causing Friedreich ataxia impede transcription elongation. EMBO Mol. Med. *2*, 120–129.

21. Butler, J.S., and Napierala, M. (2015). Friedreich's ataxia–a case of aberrant transcription termination? Transcription *6*, 33–36.

22. Al-Mahdawi, S., Pinto, R.M., Ismail, O., Varshney, D., Lymperi, S., Sandi, C., Trabzuni, D., and Pook, M. (2008). The Friedreich ataxia GAA repeat expansion mutation induces comparable epigenetic changes in human and transgenic mouse brain and heart tissues. Hum. Mol. Genet. *17*, 735–746.

23. Kumari, D., Biacsi, R.E., and Usdin, K. (2011). Repeat expansion affects both transcription initiation and elongation in friedreich ataxia cells. J. Biol. Chem. *286*, 4209–4215.

24. Li, Y., Lu, Y., Polak, U., Lin, K., Shen, J., Farmer, J., Seyer, L., Bhalla, A.D., Rozwadowska, N., Lynch, D.R., et al. (2015). Expanded GAA repeats impede transcription elongation through the FXN gene and induce transcriptional silencing that is restricted to the FXN locus. Hum. Mol. Genet. *24*, 6932–6943.

25. Mirkin, S.M., and Frank-Kamenetskii, M.D. (1994). H-DNA and related structures. Annu. Rev. Biophys. Biomol. Struct. *23*, 541–576.

26. Bernués, J., Beltrán, R., Casasnovas, J.M., and Azorín, F. (1989). Structural polymorphism of homopurine–homopyrimidine sequences: the secondary DNA structure adopted by a d(GA.CT)22 sequence in the presence of zinc ions. EMBO J. *8*, 2087–2094.

27. Bernués, J., Beltrán, R., Casasnovas, J.M., and Azorín, F. (1990). DNA-sequence and metal-ion specificity of the formation of *H-DNA. Nucleic. Acids. Res. *18*, 4067–4073.

28. Matos-Rodrigues, G., van Wietmarschen, N., Wu, W., Tripathi, V., Koussa, N.C., Pavani, R., Nathan, W.J., Callen, E., Belinky, F., Mohammed, A., et al. (2022). S1-END-seq reveals DNA secondary structures in human cells. Mol. Cell *82*, 3538–3552.

29. Lynch, D.R., Schadt, K., Kichula, E., McCormack, S., and Lin, K.Y. (2021). Friedreich Ataxia: Multidisciplinary Clinical Care. J. Multidiscip. Healthc. *14*, 1645–1658.

30. Profeta, V., McIntyre, K., Wells, M., Park, C., and Lynch, D.R. (2023). Omaveloxolone: an activator of Nrf2 for the treatment of Friedreich ataxia. Expet Opin. Invest. Drugs *32*, 5–16.

31. Herman, D., Jenssen, K., Burnett, R., Soragni, E., Perlman, S.L., and Gottesfeld, J.M. (2006). Histone deacetylase inhibitors reverse gene silencing in Friedreich's ataxia. Nat. Chem. Biol. *2*, 551–558.

32. Soragni, E., Miao, W., Iudicello, M., Jacoby, D., De Mercanti, S., Clerico, M., Longo, F., Piga, A., Ku, S., Campau, E., et al. (2014). Epigenetic therapy for Friedreich ataxia. Ann. Neurol. *76*, 489–508.

33. Libri, V., Yandim, C., Athanasopoulos, S., Loyse, N., Natisvili, T., Law, P.P., Chan, P. K., Mohammad, T., Mauri, M., Tam, K.T., et al. (2014). Epigenetic and neurological effects and safety of high-dose nicotinamide in patients with Friedreich's ataxia: an exploratory, open-label, dose-escalation study. Lancet (London, England) *384*, 504–513.

34. Ouellet, D.L., Cherif, K., Rousseau, J., and Tremblay, J.P. (2017). Deletion of the GAA repeats from the human frataxin gene using the CRISPR-Cas9 system in YG8R-derived cells and mouse models of Friedreich ataxia. Gene Ther. *24*, 265–274.

35. Li, Y., Li, J., Wang, J., Lynch, D.R., Shen, X., D, R.C., Parekh, D., Bhat, B., Woo, C., Cherry, J.J., et al. (2021). Targeting 3' and 5' untranslated regions with antisense oligonucleotides to stabilize frataxin mRNA and increase protein expression. Nucleic Acids Res. *49*, 11560–11574.

36. Li, L., Shen, X., Liu, Z., Norrbom, M., Prakash, T.P., O'Reilly, D., Sharma, V.K., Damha, M.J., Watts, J.K., Rigo, F., and Corey, D.R. (2018). Activation of Frataxin Protein Expression by Antisense Oligonucleotides Targeting the Mutant Expanded Repeat. Nucleic Acid Therapeut. *28*, 23–33.

37. Li, L., Matsui, M., and Corey, D.R. (2016). Activating frataxin expression by repeat-targeted nucleic acids. Nat. Commun. *7*, 10606.

38. Kilikevicius, A., Wang, J., Shen, X., Rigo, F., Prakash, T.P., Napierala, M., and Corey, D.R. (2022). Difficulties translating antisense-mediated activation of Frataxin expression from cell culture to mice. RNA Biol. *19*, 364–372.

39. Bergquist, H., Rocha, C.S.J., Álvarez-Asencio, R., Nguyen, C.H., Rutland, M.W., Smith, C.I.E., Good, L., Nielsen, P.E., and Zain, R. (2016). Disruption of Higher Order DNA Structures in Friedreich's Ataxia (GAA)n Repeats by PNA or LNA Targeting. PLoS One *11*, e0165788.

40. Bergquist, H., Nikravesh, A., Fernández, R.D., Larsson, V., Nguyen, C.H., Good, L., and Zain, R. (2009). Structure-specific recognition of Friedreich's ataxia (GAA)n repeats by benzoquinoquinoxaline derivatives. Chembiochem *10*, 2629–2637.

41. Rastokina, A., Cebrián, J., Mozafari, N., Mandel, N.H., Smith, C.I.E., Lopes, M., Zain, R., and Mirkin, S.M. (2023). Large-scale expansions of Friedreich's ataxia GAATTC repeats in an experimental human system: role of DNA replication and prevention by LNA-DNA oligonucleotides and PNA oligomers. Nucleic acids research *51*, 8532–8549.

42. Petersen, M., Bondensgaard, K., Wengel, J., and Jacobsen, J.P. (2002). Locked nucleic acid (LNA) recognition of RNA: NMR solution structures of LNA:RNA hybrids. J. Am. Chem. Soc. *124*, 5974–5982.

43. Moreno, P.M.D., Geny, S., Pabon, Y.V., Bergquist, H., Zaghloul, E.M., Rocha, C.S.J., Oprea, I.I., Bestas, B., Andaloussi, S.E., Jørgensen, P.T., et al. (2013). Development of bis-locked nucleic acid (bisLNA) oligonucleotides for efficient invasion of supercoiled duplex DNA. Nucleic Acids Res. *41*, 3257–3273.

44. Saher, O., Zaghloul, E.M., Umek, T., Hagey, D.W., Mozafari, N., Danielsen, M.B., Gouda, A.S., Lundin, K.E., Jørgensen, P.T., Wengel, J., et al. (2023). Chemical Modifications and Design Influence the Potency of Huntingtin Anti-Gene Oligonucleotides. Nucleic Acid Therapeut. *33*, 117–131.

45. Hori, S.I., Yamamoto, T., Waki, R., Wada, S., Wada, F., Noda, M., and Obika, S. (2015). Ca2+ enrichment in culture medium potentiates effect of oligonucleotides. Nucleic Acids Res. *43*, e128.

46. Dürr, A., Cossee, M., Agid, Y., Campuzano, V., Mignard, C., Penet, C., Mandel, J.L., Brice, A., and Koenig, M. (1996). Clinical and genetic abnormalities in patients with Friedreich's ataxia. N. Engl. J. Med. *335*, 1169–1175.

47. Voloshin, O.N., Mirkin, S.M., Lyamichev, V.I., Belotserkovskii, B.P., and Frank-Kamenetskii, M.D. (1988). Chemical probing of homopurine-homopyrimidine mirror repeats in supercoiled DNA. Nature *333*, 475–476.

48. Grabczyk, E., and Usdin, K. (2000). The GAA*TTC triplet repeat expanded in Friedreich's ataxia impedes transcription elongation by T7 RNA polymerase in a length and supercoil dependent manner. Nucleic Acids Res. *28*, 2815–2822.

49. Lundin, K.E., Højland, T., Hansen, B.R., Persson, R., Bramsen, J.B., Kjems, J., Koch, T., Wengel, J., and Smith, C.I.E. (2013). Biological activity and biotechnological aspects of locked nucleic acids. Adv. Genet. *82*, 47–107.

50. Brown, A.F., Parkinson, M.H., Garcia-Moreno, H., Mudanohwo, E., Labrum, R., Sweeney, M., and Giunti, P. (2021). Friedreich's Ataxia Frequency in a Large Cohort of Genetically Undetermined Ataxia Patients. Front. Neurol. *12*, 736253.

51. Huichalaf, C., Perfitt, T.L., Kuperman, A., Gooch, R., Kovi, R.C., Brenneman, K.A., Chen, X., Hirenallur-Shanthappa, D., Ma, T., Assaf, B.T., et al. (2022). In vivo overexpression of frataxin causes toxicity mediated by iron-sulfur cluster deficiency. Mol. Ther. Methods Clin. Dev. *24*, 367–378.

52. Kaihatsu, K., Janowski, B.A., and Corey, D.R. (2004). Recognition of chromosomal DNA by PNAs. Chem. Biol. *11*, 749–758.

53. Lin, M., and Guo, J.T. (2019). New insights into protein-DNA binding specificity from hydrogen bond based comparative study. Nucleic Acids Res. *47*, 11103–11113.

54. Zaghloul, E.M., Gissberg, O., Moreno, P.M.D., Siggens, L., Hällbrink, M., Jørgensen, A.S., Ekwall, K., Zain, R., Wengel, J., Lundin, K.E., and Smith, C.I.E. (2017). CTG repeat-targeting oligonucleotides for down-regulating Huntingtin expression. Nucleic Acids Res. *45*, 5153–5169.

55. Umek, T., Olsson, T., Gissberg, O., Saher, O., Zaghloul, E.M., Lundin, K.E., Wengel, J., Hanse, E., Zetterberg, H., Vizlin-Hodzic, D., et al. (2021). Oligonucleotides Targeting DNA Repeats Downregulate Huntingtin Gene Expression in Huntington's Patient-Derived Neural Model System. Nucleic Acid Therapeut. *31*, 443–456.

56. Zain, R., and Smith, C.I.E. (2019). Targeted Oligonucleotides for Treating Neurodegenerative Tandem Repeat Diseases. Neurotherapeutics *16*, 248–262.

57. Zanella, I., Derosas, M., Corrado, M., Cocco, E., Cavadini, P., Biasiotto, G., Poli, M., Verardi, R., and Arosio, P. (2008). The effects of frataxin silencing in HeLa cells are rescued by the expression of human mitochondrial ferritin. Biochim. Biophys. Acta. *1782*, 90–98.

OMTN, Volume *36*

Supplemental information

Anti-gene oligonucleotides targeting Friedreich's ataxia expanded GAA·TTC repeats increase Frataxin expression

Negin Mozafari, Salomé Milagres, Tea Umek, Cristina S.J. Rocha, Claudia M. Vargiu, Fiona Freyberger, Osama Saher, Marek Napierala, Jill S. Napierala, Pontus Blomberg, Per T. Jørgensen, Tanel Punga, C. I. Edvard Smith, Jesper Wengel, and Rula Zain

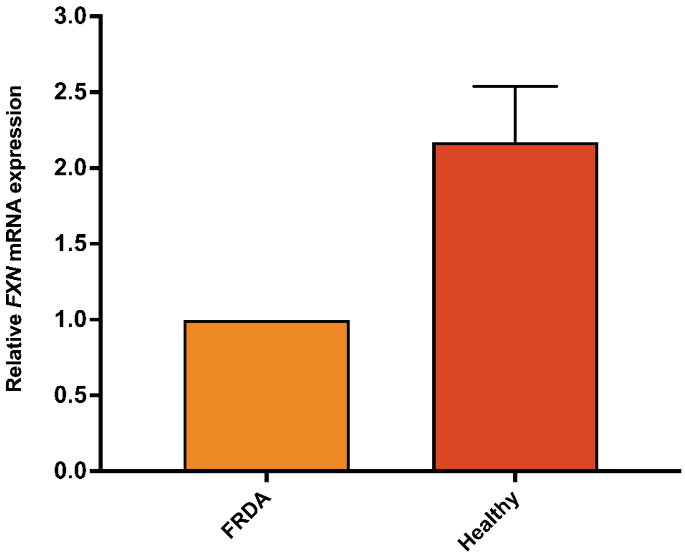

Figure S1. *FXN* mRNA levels of GM03816 (FRDA) and GM08402 (healthy) fibroblasts were assessed by RT-qPCR. The values were normalized to *HPRT1* levels as reference gene and the level of *FXN* mRNA in FRDA cells was set to one. Results are presented as Mean ±SD (n=3). Difference between means (healthy-FRDA) ± SD =1.171 ± 0.2117.

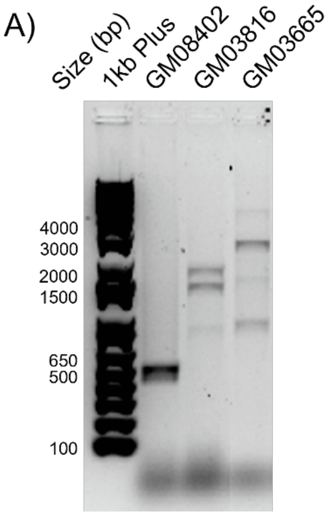

B)

Cell line	Repeats	Expected bp	Expected bp + flanking region
**GM08402**	Healthy	-	“580-580”
**GM03816**	330-380	990-1140	1570 - 1720
**GM03665**	780-1410	2340-4230	2920-4900

Figure S2. *FXN* gene GAA•TTC repeat length in primary fibroblasts used in this study. Genomic DNA was extracted from GM08402, GM03816 and GM03665 patient derived fibroblasts and the GAA•TTC repeats at the *FXN* locus were amplified by PCR. **A)** Agarose gel analysis of GAA•TTC repeat size in GM08402 (healthy) and GM03816 and GM03665 (FRDA) fibroblasts. **B)** GAA•TTC repeat length and expected size after PCR amplification.

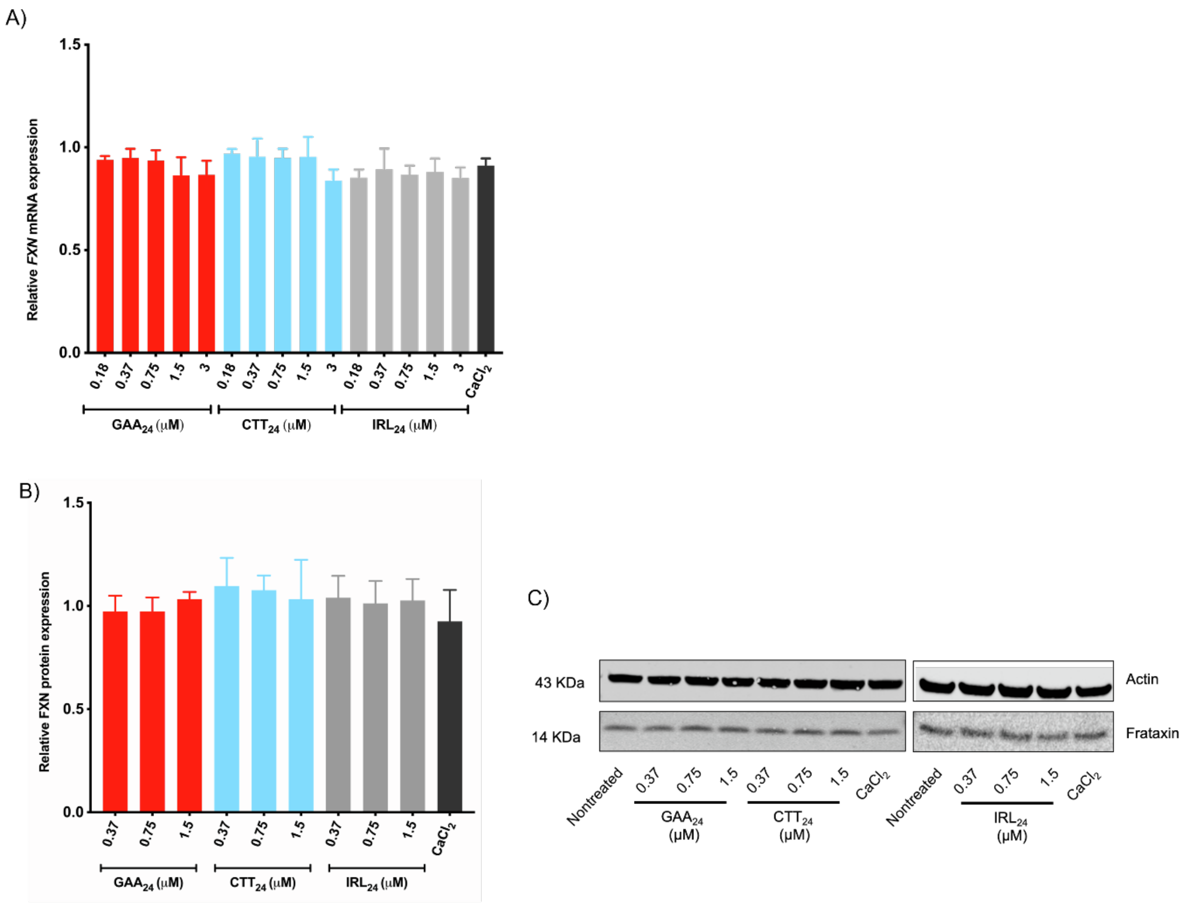

Figure S3. A-GOs do not affect neither FXN mRNA nor protein expression in healthy fibroblasts. FXN mRNA and protein expression of 6718 healthy fibroblasts after gymnotic delivery of ONs in medium supplemented with 9 mM $CaCl_2$. The cells were harvested 4 days post-treatment. *FXN* mRNA levels were determined using RT-qPCR and *FXN* levels were normalized to *HPRT1* as a reference gene. FXN protein levels were determined using western blot and FXN was normalized to Actin levels as a reference gene. For both mRNA and protein expression, relative FXN expression is shown after normalizing to the control NT cells. Results are presented as Mean ±SD, n≥3. Statistics were performed with one-way ANOVA Multiple Comparison, (Šidák), towards control ONs. (* = $P < 0.05$, ** = $P < 0.01$, *** = $P < 0.001$).

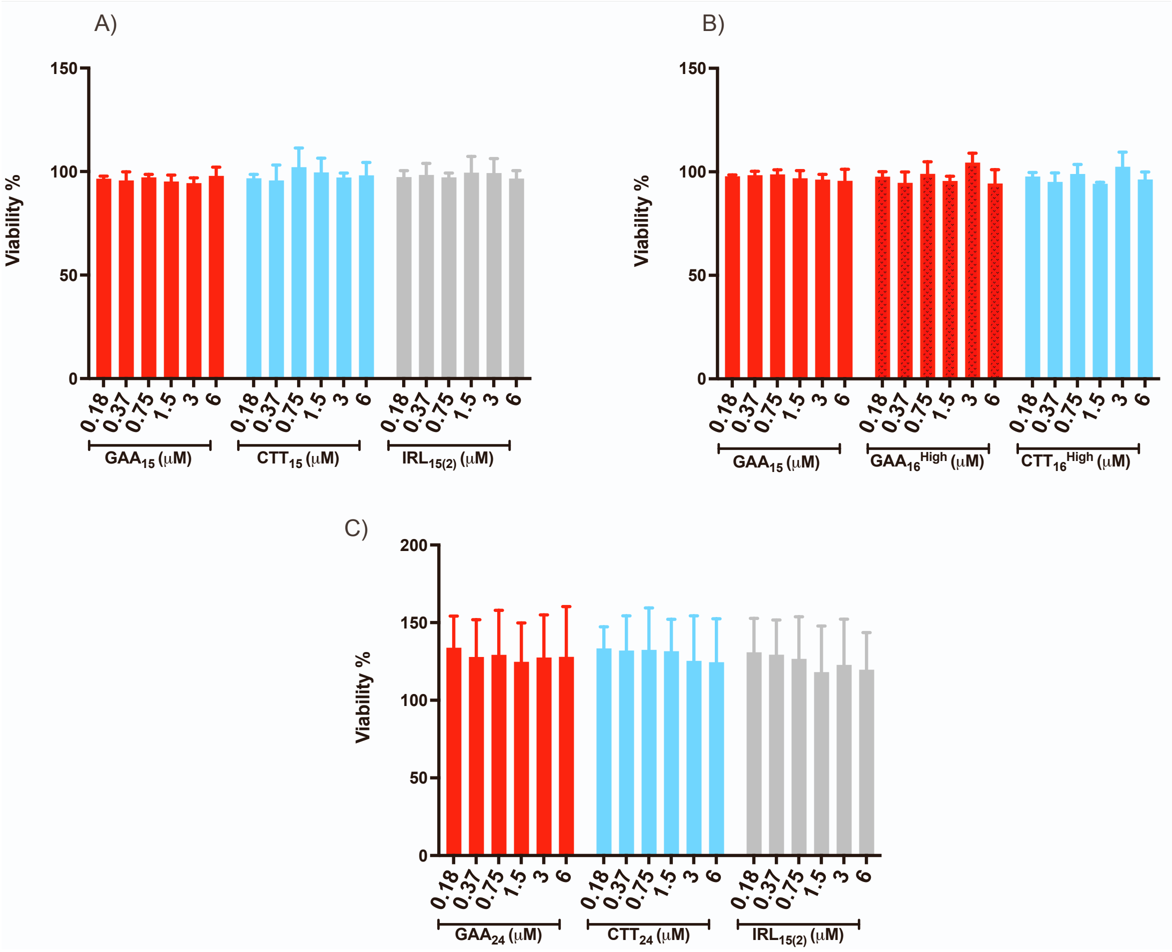

Figure S4. No significant cytotoxicity was detected after treatment of FRDA derived patient's cells with selected ONs. Viability percentage of GM03816 cells after gymnotic delivery of ONs in medium supplemented with 9 mM $CaCl_2$ throughout 48 hours treatment. The relative values were obtained by normalization of ONs treated cells versus cells in the presence of 9 mM $CaCl_2$. Results are presented as Mean ±SD, A and B (n=3) and C (n=2). Statistics were performed with two-way ANOVA Multiple Comparison (Turkey). The mean of each condition was compared to the mean of every other condition. (* = $P < 0.05$, ** = $P < 0.01$, *** = $P < 0.001$).

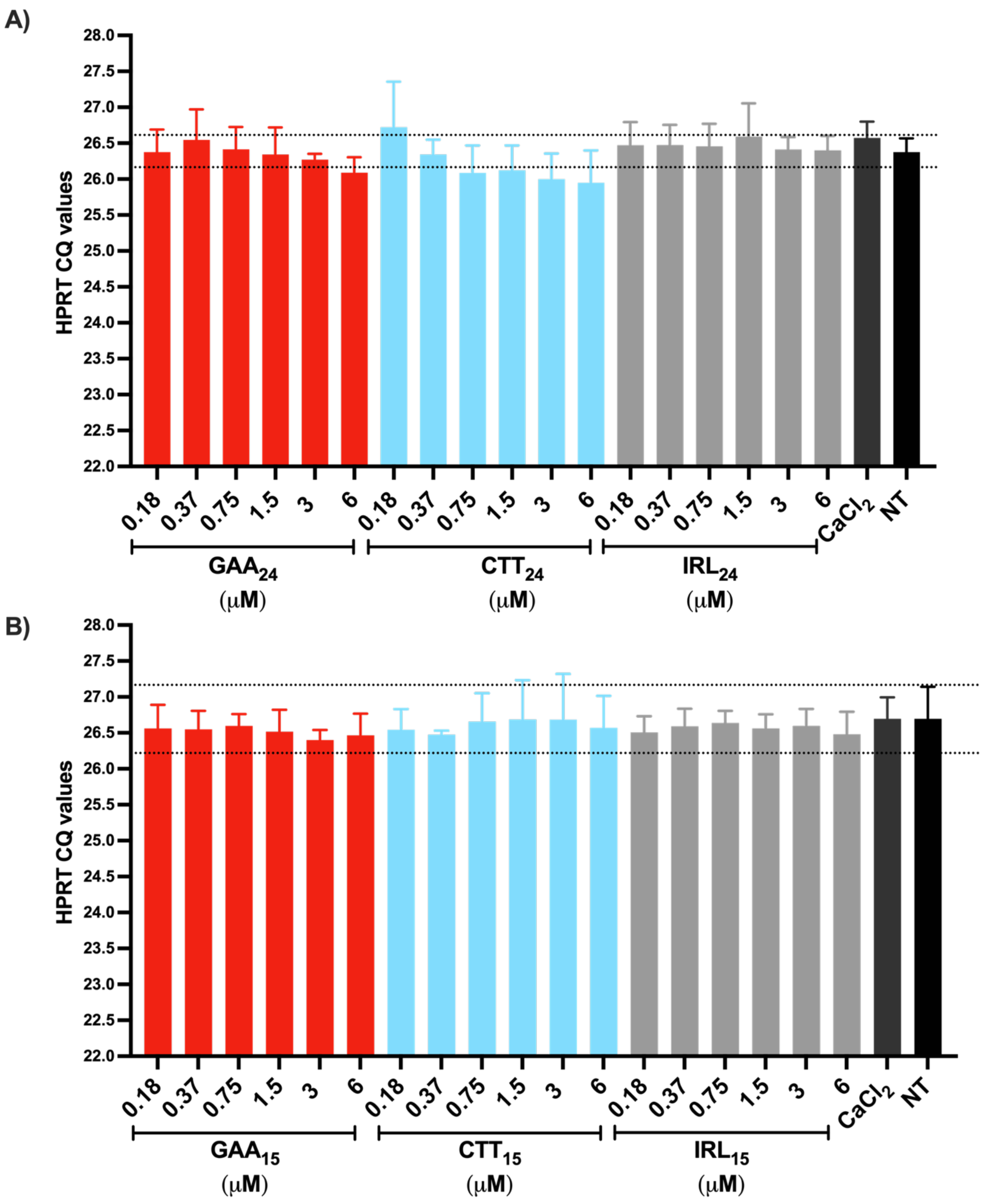

Figure S5. No significant differences in *HPRT1* C_q values were detected after treatment of FRDA derived patient's cells with selected ONs. GM03816 fibroblasts were treated with **A)** 15-mers and **B)** 24-mers GAA, CTT and IRL ONs at different doses ranging from 0.18 - 6 μM. Treated and NT cells were harvested 4 days after transfection, and *HPRT1* C_q values were assessed by RT-qPCR. Results are presented as Mean ±SD (n≥3). Statistics were performed with Kruskal-Wallis test, towards NT cells.

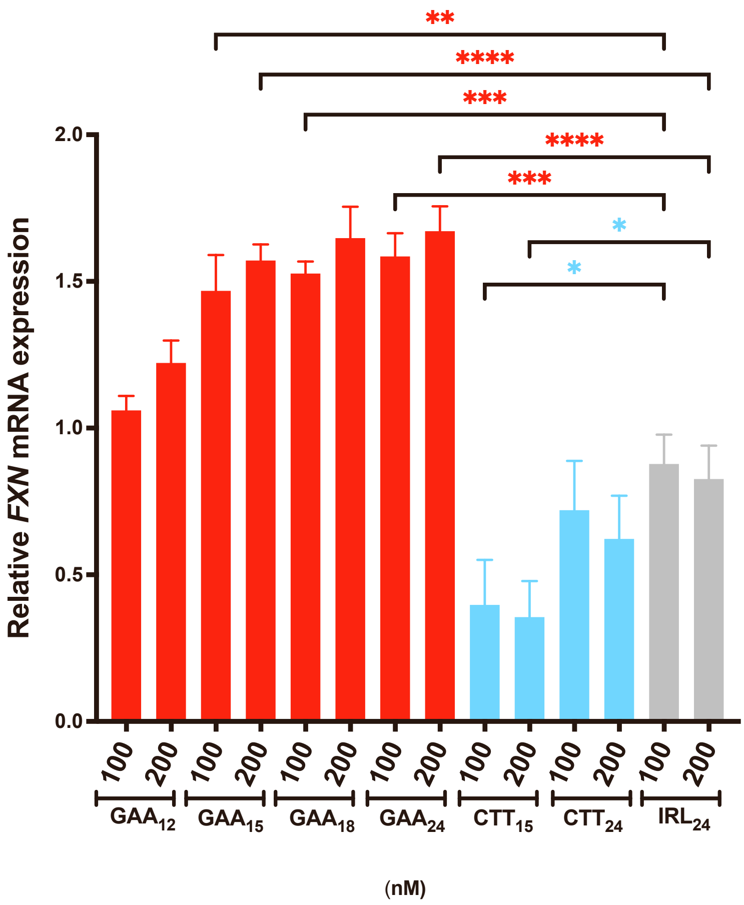

Figure S6. GAA A-GOs upregulate *FXN* mRNA expression in a FRDA cell model with a higher number of repeats. GM03665 fibroblasts were treated with different lengths of GAA, CTT and IRL ONs at 100 and 200 nM. Treated and NT cells were harvested 4 days after transfection, and *FXN* mRNA levels were assessed by RT-qPCR. The values were normalized to *HPRT1* levels as reference gene and were compared to NT cells. Results are presented as Mean with SD (n ≥3). Statistics were performed with one-way ANOVA Multiple Comparison, (Šídák), towards corresponding IRL concentration. (* = P < 0.05, ** = P < 0.01, *** = P < 0.001, **** = P < 0.0001;).

A)

Gene	Symbol	Repeats (location; number)	Binding of GAA_{24}
Adenosine Kinase	ADK	GAA:TTC (intron 6; 13)	DNA template strand
Ribosomal protein S6 kinase A5	RPS6KA5	AAG:CTT (3'UTR; 8)	DNA template strand
Lysine acetyltransferase 6B	KAT6B	GAA:TTC (exon 16; 8)	DNA template strand
PR/SET domain 10	PRDM10	CTT:AAG (3'UTR; 10)	mRNA
Ras And Rab Interactor 2	RIN2	CTT:AAG (intron 2; 22 & 14)	pre-mRNA

B)

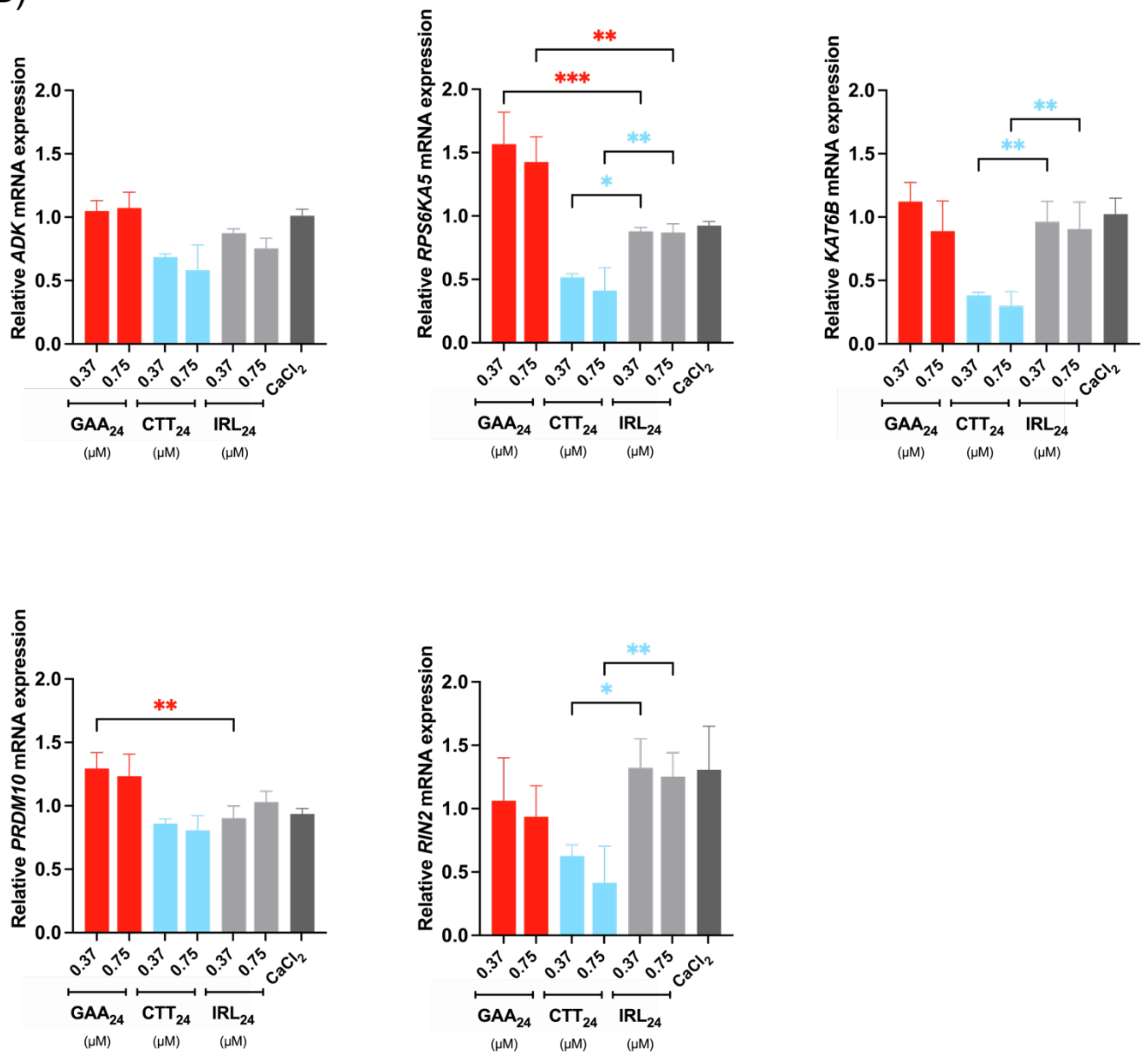

Figure S7. GAA_{24} and CTT_{24} affect the mRNA levels of other repeat-containing genes. A) Information on the selected genes for off-target analysis. **B)** 4869 fibroblasts were treated with concentrations of 0.37 and 0.75 µM, in medium supplemented with 9 mM $CaCl_2$. Four days post-treatment, the cells were harvested, and mRNA levels were analysed. The values were normalized to *HPRT* as a reference gene. The expression of NT cells without the presence of $CaCl_2$ was set to 1. Results are presented as Mean ± SD, (n = 3). Statistics were performed with one-way ANOVA Multiple Comparison (Šidák) towards the same IRL_{24} concentration (* = $P < 0.05$, ** = $P < 0.01$, *** = $P < 0.001$).

Table S1. List of all the primers and probe sets used in this study. *FXN* was multiplexed with *HPRT1*_v1, *RPS6KA5* to *KAT6B* were multiplexed with *HPRT1*_v2 and *RIN2* was multiplexed with *HPRT1*_v3. *HPRT1*_v1, *FXN* and *HPRT1*_v3 sets were purchased as DNA oligos and dual-labelled probes from Merck. *HPRT1*_v2, *RPS6KA5*, *PRDM10*, *ADK*, *KAT6B* and *RIN2* were purchased as PrimeTime™ qPCR Probe Assays from IDT. 6FAM, 56FAM, 5HEX and HEX are fluorophores. BHQ1, 3IABkFQ and ZEN are quenchers.

Gene	Sequence (5'-3')	
***HPRT1*_v1**	Fw primer	AGGGATTTGAATCATGTTTG
	Rv primer	CGATGTCAATAGGACTCC
	Probe	/6FAM/ACTCAACTTGAACTCTCATCTTAGGCT/BHQ1/
FXN	Fw primer	GTGGAGATCTAGGAACCTATG
	Rv primer	TTAAGGCTTTAGTGAGCTCTG
	Probe	/HEX/TCCAGTCATAACGCTTAGGTCCAC/BHQ1/
***HPRT1*_v2**	Fw primer	AGGATTTGGAAAGGGTGTTTATTC
	Rv primer	CCCATCTCCTTCATCACATCTC
	Probe	/56-FAM/ATGGACAGG/ZEN/ACTGAACGTCTTGCT/3IABkFQ/
RPS6KA5	Fw primer	CATGCTGAGAAGGTGGGAATAG
	Rv primer	GGCATACAGCTTTCCAGTATCA
	Probe	/5HEX/TTGAGCTCC/ZEN/TGAAGGTCCTAGGAACT/3IABkFQ/
PRDM10	Fw primer	GGACACAGGACTTGCTACATT
	Rv primer	GGATTGGGATAGTGGTCTGTTC
	Probe	/5HEX/ACTCTGAAT/ZEN/GGGCTGGATCAACCA/3IABkFQ/
ADK	Fw primer	GCTGAAGACAAACACAAGGAAC
	Rv primer	TCATCCACTGAGCCACTTTAAT
	Probe	/5HEX/ATGCTGGTG/ZEN/GCTCTACCCAGAATT/3IABkFQ/
KAT6B	Fw primer	GACAAACAGAGGAAGAGGAAGG
	Rv primer	CACTTTAGAGGTTCGGGATTGT
	Probe	/5HEX/TGCTTCAAG/ZEN/AATGCTGACCCTTGTAGA/3IABkFQ/
***HPRT1*_v3**	Fw primer	GAGCTATTGTAATGACCAGTC
	Rv primer	TGACCAAGGAAAGCAAAG
	Probe	/6FAM/TGCCAGTGTCAATTATATCTTCCACAA/BHQ1/
RIN2	Fw primer	CCACTCAAGGAATTTGCCATAAA
	Rv primer	GAAAGCAATGAGCCGGAATAAA
	Probe	/5HEX/CCCTGGAAG/ZEN/GCTCAGGAATCAGTTT/3IABkFQ/
